# Identification of a xenobiotic as a potential environmental trigger in primary biliary cholangitis

**DOI:** 10.1016/j.jhep.2018.06.027

**Published:** 2018-11

**Authors:** Philip M. Probert, Alistair C. Leitch, Michael P. Dunn, Stephanie K. Meyer, Jeremy M. Palmer, Tarek M. Abdelghany, Anne F. Lakey, Martin P. Cooke, Helen Talbot, Corinne Wills, William McFarlane, Lynsay I. Blake, Anna K. Rosenmai, Agneta Oskarsson, Rodrigo Figueiredo, Colin Wilson, George E. Kass, David E. Jones, Peter G. Blain, Matthew C. Wright

**Affiliations:** 1 Health Protection Research Unit, Wolfson Building, Institute of Cellular Medicine, Newcastle University, Newcastle Upon Tyne NE2 4AA, United Kingdom; 2 Department of Pharmacology and Toxicology, Faculty of Pharmacy, Cairo University, Kasr El-Aini St., Cairo 11562, Egypt; 3 School of Civil Engineering and Geosciences, Drummond Building, Newcastle University, Newcastle upon Tyne NE1 7RU, United Kingdom; 4 School of Chemistry, Bedson Building, Newcastle University, Newcastle upon Tyne NE1 7RU, United Kingdom; 5 Institute for Sustainability, The Key Building, Newcastle University, Newcastle upon Tyne NE4 5TQ, United Kingdom; 6 Swedish University of Agricultural Sciences, Uppsala, Sweden; 7 Freeman Hospital, Newcastle upon Tyne, Tyne and Wear NE7 7DN, United Kingdom; 8 European Food Safety Authority, Via Carlo Magno 1A, 43126 Parma, Italy

**Keywords:** Liver progenitor, Mitochondria, Cholangiocyte, Biliary disease, AHR, ERα, PPARα, Ionic solvent, C8mim, B-13, AR42J-B13

## Abstract

**Background & Aims:**

Primary biliary cholangitis (PBC) is an autoimmune-associated chronic liver disease triggered by environmental factors, such as exposure to xenobiotics, which leads to a loss of tolerance to the lipoic acid-conjugated regions of the mitochondrial pyruvate dehydrogenase complex, typically to the E2 component. We aimed to identify xenobiotics that might be involved in the environmental triggering of PBC.

**Methods:**

Urban landfill and control soil samples from a region with high PBC incidence were screened for xenobiotic activities using analytical, cell-based xenobiotic receptor activation assays and toxicity screens.

**Results:**

A variety of potential xenobiotic classes were ubiquitously present, as identified by their interaction with xenobiotic receptors – aryl hydrocarbon receptor, androgen receptor and peroxisome proliferator activated receptor alpha – in cell-based screens. In contrast, xenoestrogens were present at higher levels in soil extracts from around an urban landfill. Furthermore, two landfill sampling sites contained a chemical(s) that inhibited mitochondrial oxidative phosphorylation and induced the apoptosis of a hepatic progenitor cell. The mitochondrial effect was also demonstrated in human liver cholangiocytes from three separate donors. The chemical was identified as the ionic liquid [3-methyl-1-octyl-1*H*-imidazol-3-ium]^+^ (M8OI) and the toxic effects were recapitulated using authentic pure chemical. A carboxylate-containing human hepatocyte metabolite of M8OI, bearing structural similarity to lipoic acid, was also enzymatically incorporated into the E2 component of the pyruvate dehydrogenase complex via the exogenous lipoylation pathway *in vitro*.

**Conclusions:**

These results identify, for the first time, a xenobiotic in the environment that may be related to and/or be a component of an environmental trigger for PBC. Therefore, further study in experimental animal models is warranted, to determine the risk of exposure to these ionic liquids.

**Lay summary:**

Primary biliary cholangitis is a liver disease in which most patients have antibodies to mitochondrial proteins containing lipoic acid binding site(s). This paper identified a man-made chemical present in soils around a waste site. It was then shown that this chemical was metabolized into a product with structural similarity to lipoic acid, which was capable of replacing lipoic acid in mitochondrial proteins.

## Highlights

• Soil samples around landfill waste sites were screened for their ability to activate xenobiotic receptors and toxicity.
• Xenoestrogens were present at higher levels in soils around a landfill site.
• An ionic liquid was found to be present at high levels in two soil sampling sites near landfill.
• The ionic liquid inhibited cellular oxidative phosphorylation, was toxic to a liver progenitor cell line and induced apoptosis.
• The ionic liquid was metabolised by human hepatocytes to a carboxylic acid that bore structural similarity to lipoic acid.

## Graphical abstract

**Figure:**
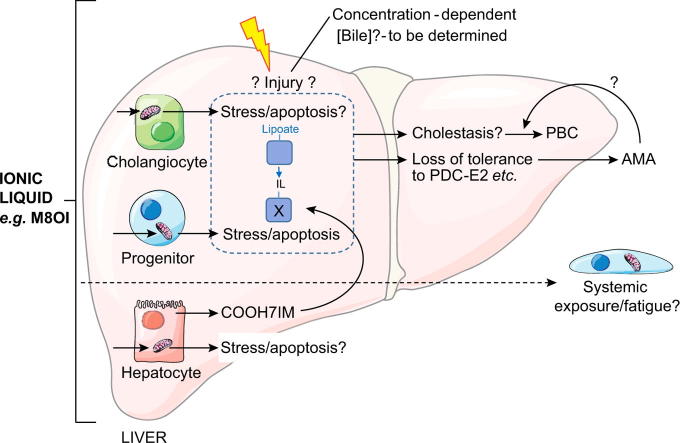

## Introduction

Liver disease constitutes the third most common cause of premature death in the UK, with an upward trend in mortality.[1], [2] The major causes of liver disease such as obesity, viral infection and chronic alcohol consumption are preventable. However, the causes of rarer types of liver disease – such as primary biliary cholangitis (PBC) and primary sclerosing cholangitis (PSC) are unknown and as a consequence, prevention and/or treatments are limited.^3^ A variety of factors have been linked to increased incidence of PBC and PSC in populations. In both cases, although there is a clear genetic pre-disposition,[4], [5] there is also evidence that environmental factors – such as exposure to foreign compounds (xenobiotics) – determine the likelihood of developing disease.[6], [7], [8], [9]

In the majority of cases, PBC is associated with an immunological loss of tolerance to the lipoic acid-conjugated regions of the mitochondrial pyruvate dehydrogenase complex (PDC), also known as dihydrolipoamide-S-acetyl transferase, leading to the presence of high levels of diagnostic anti-mitochondrial antibodies (AMA) in patient sera.[10], [11] Thus, one potential trigger for PBC may be exposure to chemicals that structurally and chemically mimic lipoic acid and may therefore be capable of being enzymatically incorporated into the E2 component of PDC (PDC-E2) in place of lipoic acid.[10], [11] Specific disease pathology in the liver (since the antigen for AMA is present in most cells of the body) is thought to be due to selective exposure of the neo-antigen (*e.g.* via selective apoptosis of hepatic intrahepatic duct cells/cholangiocytes).^12^

A number of signaling pathways have been identified in cells that function to detect xenobiotics and modulate gene expression in order to facilitate their metabolism and excretion. Polycyclic aromatic hydrocarbons (PAHs) are environmental organic xenobiotics consisting of two or more clustered benzene rings, which are present in crude oil and coal tar and contaminate the environment through fossil fuel burning, incineration of waste and other industrial processes.^13^ They are hydrophobic and often persist in the environment and tissues. Many PAHs are suspected or known to be toxic, genotoxic and carcinogenic.^14^ The aryl hydrocarbon receptor (AHR) binds and is transcriptionally activated by many PAHs.^15^ In the liver, this leads to the increased expression of a variety of genes associated with xenobiotic metabolism (*e.g.* the *AHR* locus).^15^ However, the function of the AHR likely extends beyond xenobiotic metabolism alone as its endogenous functions appear to include roles in the immune system.^16^ In the thymus, AHR activation can lead to thymocyte apoptosis and thymic atrophy.^17^

The peroxisome proliferator activated receptor alpha (PPARα) is a nuclear receptor that functions in the regulation of fatty acid oxidation. In addition to its activation by selected endogenous lipids, the receptor is also activated by fibrate drugs (its pharmacological target) and xenobiotics such as polyhalogenated chemicals *e.g.* perfluorooctane sulfonate.^18^ The nuclear estrogen receptor alpha (ERα) appears to be a frequent target for a variety of natural (*e.g.* plant phytoestrogens) and xenobiotic man-made chemicals (*e.g.* pesticides). It has been proposed that xenoestrogens are responsible for a spectrum of adverse effects in wildlife and man that include malformations in the male genital tract; decreased sperm quality; neuroendocrinological, behavioral and metabolic effects and cancer.[19], [20], [21]

In an attempt to identify potential environmental xenobiotic triggers, urban landfill and control soil samples from a region with high PBC incidence were screened for xenobiotic activities using a variety of *in vitro* cell-based assays.

## Materials and methods

### Chemicals

3-Methyl-1-octyl-1H-imidazol-3-ium (M8OI) was purchased from Sigma (Poole, UK). 1-(8-Hydroxyoctyl)-3-methyl-imidazolium (HO8IM) and 1-(7-carboxyheptyl)-3-methyl-1H-imidazol-3-ium (COOH7IM) were custom synthesized with purity and chemical structures determined by high-performance liquid chromatography (HPLC), mass spectrometry and nuclear magnetic resonance techniques (NMR) (for COOH7IM, see Fig. S11).

### Preparation of soil extracts

Surface soil samples (0–5 cm in depth) were collected and extraneous vegetable matter and stones removed. Each sample was divided into four 250 g portions. A sample of one portion was digested using *aqua regia* in accordance with BS7755 for metals analysis. Two portions were subjected to either methanol (for polar molecule) or chloroform (for hydrophobic chemical) extractions by sonicating with 300 ml of solvent for 10 min, followed by addition of a further 100 ml of solvent and sonication for a further 10 min prior to filtration with 25 µm filters and collection of filtrate. Filtrates were evaporated in a rotary evaporator and then blown down to near dryness under a stream of nitrogen. The methanol extracted material was divided into two and added to either 10 ml of phosphate buffered saline (PBS, 137 mM NaCl, 27 mM KCl, 100 mM phosphate pH 7.4) or 10 ml of ethanol. The chloroform extracted material was re-dissolved into 10 ml of chloroform. The solvated extracted chemicals were then separated from any precipitate and stored at −20 °C (ethanol and chloroform extracts) or 4 °C (PBS extracts).

Thirteen soil samples were collected from allotments, footpaths and the roadside verges surrounding an urban landfill site. Three control soil samples were collected from three separate sites. One sample was obtained from the University farm in rural Northumberland at a site with controlled fertilizer regime for the last 130 years. The remaining two control samples were obtained from gardens in urban areas in the region.

### Cell culture

Rat B-13 hepatocyte progenitor cells were routinely expanded in low glucose (1000 mg/l) Dulbecco’s minimum essential medium (DMEM) supplemented with 10% (v/v) fetal calf serum (FCS), 80 u/ml penicillin and 80 µg/ml streptomycin. B-13 cells were converted into functional hepatocytes (B-13/H cells) *in vitro* through addition of 10 nM dexamethasone, essentially as previously outlined.[52], [53], [23] B-13/H cells are a non-proliferative functional hepatocyte-like cell expressing a variety of hepatic functions (such as functional cytochrome P450s) at near normal liver levels.^54^ The human H69 cholangiocyte cell line^55^ was routinely expanded in 3:1 (v/v) ratio of DMEM and Nutrient F12 Ham’s medium supplemented with 180 µM adenine, 2 nM triiodothyronine, 5.5 µM epinephrine, 1 µM hydrocortisone, 10% v/v FCS, 1× insulin/transferrin/selenium (Gibco) and 1× Pen/Strep (Lonza). The human hepatoma HepG2 cell line was cultured as previously described.^56^ The human breast cancer MCF-7 cell line was cultured as previously described.^57^ All cells were incubated at 37 °C in a humidified incubator gassed with 5% CO_2_ in air. Human cholangiocytes were isolated from resected human liver using an immune-bead approach as previously described and cultured in 1:1 [v/v] DMEM:Hams F12 medium supplemented with 10% (v/v) FBS, 2 mM glutamine, 100 IU/ml penicillin, 100 μg/ml streptomycin, 10 ng/ml epidermal growth factor (EGF), 0.248 IU/ml insulin, 2 µg/ml hydrocortisone, 10 ng/ml cholera toxin, 2 nM tri-iodo-l-thyronine and 5 ng/ml hepatocyte growth factor (HGF).^58^ Human hepatocytes were isolated from a 42-year-old male donor by collagenase perfusion, essentially as previously described,^59^ and cultured on collagen-coated plates in Williams medium E supplemented with 10% (v/v) FCS, 80 IU/ml penicillin, 80 µg/ml streptomycin, 10 nM dexamethasone and 1 μg/ml insulin. After an overnight culture period, the medium was aspirated, the cells were washed three times with sterile PBS prior to incubation with M8OI in a short-term simplified incubation medium (STIM buffer: 0.10 M NaCl, 5.4 mM KCl, 0.34 mM Na_2_HPO_4_ 12H_2_O, 0.44 mM KH2PO4, 20 mM glucose, 1 mM CaCl_2_, 40 mM NaHCO_3_, 4 mM glutamine, 100 µM l-alanine, 100 µM l-asparagine, 100 µM l-aspartic acid, 100 µM l-glutamic acid, 100 µM glycine, 100 µM l-proline and 100 µM l-serine, pH 7.4 when gassed with 5% CO_2_ in air) to minimize interference with M8OI detection, typically 1.5 ml/well of a 6-well plate. As a control, M8OI was incubated identically in a cell-free culture vessel. After 24 h, the STIM incubation was removed, centrifuged at 13,000 rpm for 1 min and 10 volumes of supernatant clear of any cellular material retained and added to 1 volume 1% phosphoric acid. Acid-precipitated material was removed by centrifugation (13,000 rpm, 1 min) and the supernatant was retained and stored at 4 °C prior to analysis. Human tissue was obtained with patient consent and with approval of the Newcastle & North Tyneside 2 Research Ethics Committee.

### Animal study

A study with mice detailed in the supplementary data section was performed under a UK Home Office license with Local Ethics Committee approval. Animals (up to five per cage) were housed in Maxiseal 420 cm^2^ mouse cages (Arrowmight, Hereford, UK) in an enriched environment (nesting material, chew sticks and cardboard tubes) and were provided with food (RM3 Special Diet Services, UK) and water *ad libitum* in an air-conditioned environment on a 12 h light/dark cycle with regulated humidity (50% ± 10%) and temperature (23 °C ± 1 °C). All animals received humane care and the study was in compliance with institutional and ARRIVE guidelines.

For further details regarding the materials and methods used, please refer to the CTAT table and supplementary information, [60], [61], [62], [63], [64], [65], [66], [67].

## Results

### Similar levels of heavy metals, polyaromatic hydrocarbons and pesticides were present in landfill and control site soils

Soil samples were taken from around a currently active peri-urban landfill site (and from three separate control sampling sites), with all sites positioned upon an area of historic coal mining. Extracts obtained using different solvents – from aqueous to hydrophobic - were prepared and tested as schematically outlined (Fig. S1A). Analysis of both sonicated soil extracts and the *aqua regia* digests indicated low levels of contamination with a wide range of potentially toxic elements (Table S1). At all sample sites, values were typical of an urban soil lacking overt contamination.^22^ These data were supported by a metallothionein luciferase reporter gene assay screen, which indicated low levels of metals in all soil extracts (Fig. S1B). Since the landfill and control sites were also situated in a region with a history of intensive coal mining, the soil levels of common PAHs were also examined. The evidence indicates that levels were not significantly different although overall, the mean levels were higher in control soils (65 ± 54 mg/kg soil) compared to landfill soils (13 ± 19 mg/kg soil) (Table S2). A screen for 24 different pesticides indicated that 22 out of 24 were detectable in at least one soil sample and a minimum of at least four different pesticides (landfill soil sample 2) were detectable in each soil sample (Table S3). In all samples, the levels were low with only two pesticides - Diuron and Omethoate – present at levels greater than 1 ppm in any soil extract (the former, at mean higher levels in control soils compared to soils in close proximity to the landfill site). The combined mean pesticide levels were higher in control soils (total of 3.9 ± 1.81 ppm) compared to soils in close proximity to the landfill site (total of 2.0 ± 1.69 ppm).

To screen for the presence of acutely toxic chemicals, extracts were screened for toxicity in hepatic progenitor B-13, hepatocyte-like B-13/H and H69 cholangiocyte cell lines^23^ at an initial concentration of 1% (v/v) in culture media. With the exception of PBS solvated extracts from two landfill sampling sites and B-13 cells – none of the extracts were markedly toxic (Fig. S2). To reduce the potential that toxic effects might interfere with signaling pathway interactions, a further 10-fold dilution of soil extracts was routinely examined (*i.e.* 0.1% (v/v) in culture media). We demonstrate that extracts from both the landfill sampling sites and control sites induced several hundred-fold increases in AHR-dependent XRE-luc reporter gene activity (Fig. S3A), with higher fold induction observed in extracts solvating more hydrophobic chemicals (*i.e.* chloroform extracts). These data were confirmed by similar robust induction in AHR-regulated *Cyp1a1* mRNA expression in metabolically-active xenobiotic metabolizing B-13/H cells (Fig. S3B); a dose-response effect in all selected tested extracts (Fig. S3C) and induction of Cyp1a1 protein expression (Fig. S3D). Similar results were also found when ethanol and chloroform extracts were examined for activators of the human PPARα (Fig. S4), demonstrating that soil extracts from both landfill and control sites contain chemicals that activate xenobiotic receptor AHR and PPARα receptors.

### Chemicals present in landfill site soils activated the human ERα, with limited effects on the human AR

**Fig. 1:**
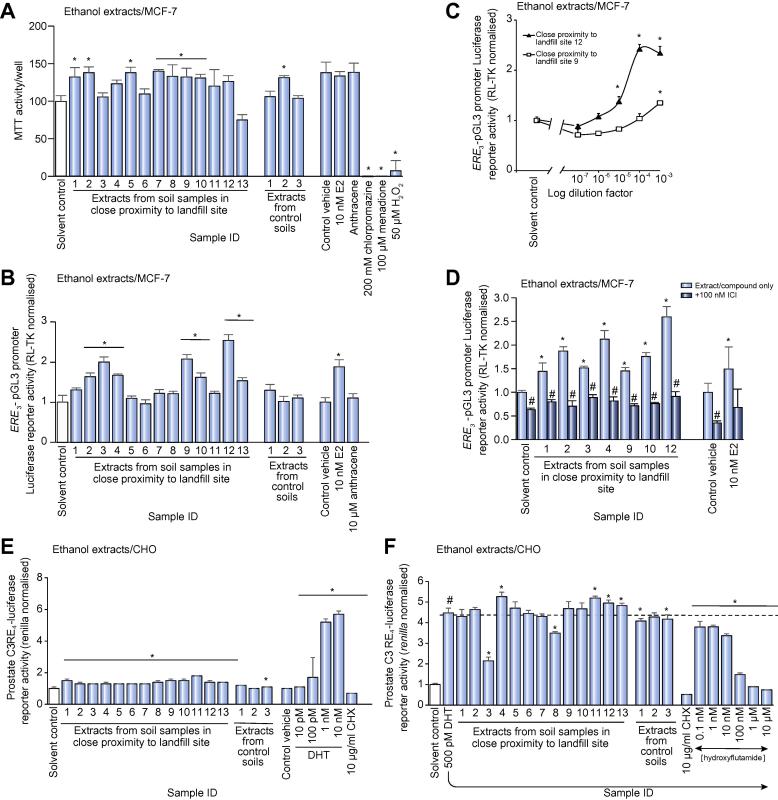
**Soil samples in close proximity to a landfill site contain biologically active levels of ERα activating chemicals.** (A) Determination of MTT reduction in MCF-7 cells following 24 h incubation with 0.1% v/v ethanol extracts. Results are expressed as the mean and SD of three separate determinations and are expressed as a percentage of 0.1% (v/v) ethanol vehicle. (B) Human ERα activation (*ERE3-*pGL3 promoter-Luc). MCF-7 cells were transfected with reporter constructs and 24 h later, treated with 0.1% v/v of the ethanol extracts. After 24 h exposure, reporter gene activities were determined as outlined in the methods section with data, the mean and SD of three separate transfections. (C) Dose-response effect for human ERα activation (*ERE3-*pGL3 promoter-Luc) in MCF-7 cells treated with the indicated dilution of landfill waste site soil ethanol extract, expressed as fold ethanol vehicle control. Data are the mean and SD of three separate determinations. (D) Human ERα activation (*ERE3-*pGL3 promoter-Luc). MCF-7 cells were transfected with reporter constructs and 18 h later, pre-treated where indicated with the ERα antagonist ICI182780) or solvent vehicle control. After 6 h, cells were then treated with 0.1% (v/v) of the indicated ethanol extracts. After 24 h exposure, reporter gene activities were determined as outlined in the methods section with data, the mean and SD of three separate transfections. *Significantly different from solvent control (for soil extracts) or control vehicle for known chemicals (*p <* 0.05) using ANOVA/Bonferroni-Holm comparison between groups or ^#^equivalent extract in the absence of ICI (*p <* 0.05) using Student’s *t* test (two tailed). (E) Human AR activation (prostate C3 RE_4_-luciferase) activation by ethanol extracts. Reporter gene activities were determined as outlined in the methods section with data, the mean and SD of at least three separate transfections. ^*^Significantly different from solvent control (for soil extracts) or control vehicle for known chemicals (*p* < 0.05) using ANOVA/Bonferroni-Holm comparison between groups. (F) Human AR activation (prostate C3 RE_4_-luciferase) antagonism by ethanol extracts after activation 500 pM DHT. Reporter gene activities were determined as outlined in the methods section with data the mean and SD of at least three separate transfections. Significantly different from ^#^solvent control or *500 pM DHT (*p <* 0.05) using ANOVA/Bonferroni-Holm comparison between groups. AR, androgren receptor; DHT, dihydrotestosterone; ER, estrogen receptor.

To determine whether soil extracts contained xenoestrogens capable of activating the human ERα, MCF-7 cells transfected with an estrogen responsive luciferase reporter gene (*ERE3-*pGL3 promoter) were exposed to soil extracts. PBS and chloroform extracts were not toxic (Fig. S5A, B) and in a minority of landfill soil samples, contained chemicals that activated the human ERα (Fig. S5D, E). However, ethanol soil extracts – which were not toxic to MCF-7 cells either (Fig. 1A), see also Fig. S5C – contained the most potent ERα activation activities, with activation restricted to several soil samples in close proximity to a landfill site (Fig. 1B). There was low to undetectable estrogenic activity detectable in ethanol extracts from the control soil samples (Fig. 1B). Clear dose-responses were observed through further dilution of ethanol extracts (Fig. 1C). Inhibition of activation by co-incubation with the anti-estrogen ICI182780 (Fig. 1D) confirmed direct ERα interaction and activation of transcriptional function by the ethanol extracts from soil samples in proximity to a landfill site. In contrast, there was limited activity directed toward the human androgen receptor (AR) with only control site 2 yielding a statistically significant increase in reporter gene expression, which is likely not to be of biological significance (Fig. 1E). Two landfill soil sites contained chemicals capable of antagonizing dihydrotestosterone activation of the human AR (Fig. 1F).

In contrast to AHR and PPARα-activating chemicals, these data demonstrate that several extracts from sites close to a landfill site contain human ERα-activating chemicals, which were not present in control soil samples.

### Two aqueous landfill soil extracts blocked hepatic progenitor cell proliferation and induced cell death

**Fig. 2:**
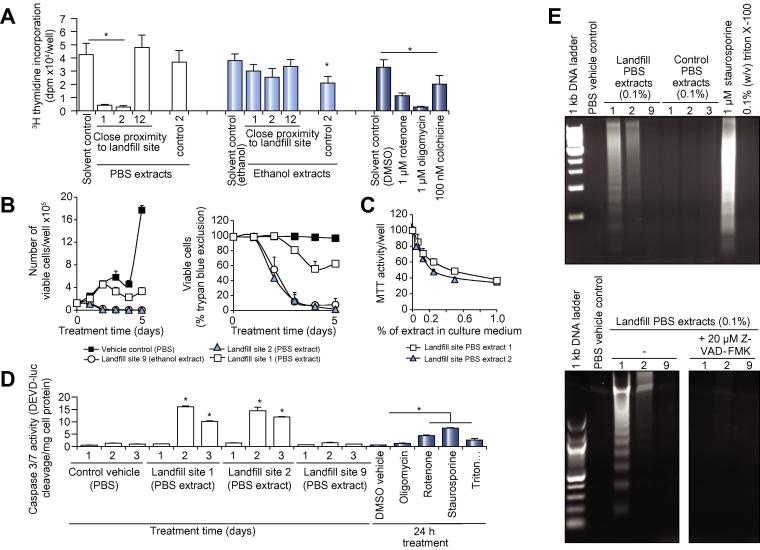
**Landfill sampling site 1 and 2 PBS extracts inhibit proliferation and induce the apoptosis of the hepatic progenitor B-13 cell.** (A) ^3^H-thymidine uptake in B-13 cells. B-13 cells were pre-treated with the indicated compounds or 1% (v/v) soil extracts for 6 h prior to addition of ^3^H-thymidine. Following an overnight exposure, ^3^H-thymidine incorporation was determined. Results are the mean and SD of six separate determinations from the same experiment typical of at least three separate experiments. *Significantly different from respective solvent control (*p <* 0.05) using ANOVA/Bonferroni-Holm comparison between groups. (B) B-13 cells were treated with the indicated environmental samples at a final concentration of 1% (v/v) for 24 h prior to a medium change. Total number (left panel) and percentage viable cells (right panel) were then determined based on ability to exclude trypan blue. Data are the mean and SD of three separate treatments for the same experiment typical of at least three separate experiments. (C) Dose-response effect of PBS extracts on MTT reduction activity in B-13 cells determined after 24 h exposure. Data are the mean and SD of three separate experiments. *Significantly different from 0%/PBS solvent control (*p <* 0.05) using ANOVA/Bonferroni-Holm comparison between groups. (D) Caspase 3/7 activity in B-13 cells treated as indicated with environmental samples at 1% (v/v) for 1–3 days or with 1 µM oligomycin, 1 µM rotenone or 1 µM staurosporine for 24 h. Following treatment, an ApoTox-glo triplex assay was used to determine caspase 3/7 activity. Results are expressed relative to the relevant vehicle treated cells and are the mean and SD of three separate determinations from the same experiment typical of at least three separate experiments. *Significantly different from solvent control (PBS) at the equivalent time point or DMSO vehicle control for known chemicals (*p <* 0.05) using ANOVA/Bonferroni-Holm comparison between groups. (E) B-13 cells were treated with 1% (v/v) extracts for two days prior to genomic DNA isolation and analysis for nucleosomal ladder formation (left panel). B-13 cells were co-treated with the caspase inhibitor Z-VAD-FMK at the time of exposure to PBS extracts as indicated (right panel), nucleosomal ladders are from the same gel imaged and processed identically. PBS, phosphate buffered saline.

PBS extracts from two sampling sites in close proximity to the landfill site showed marked toxicity in the hepatic B-13 progenitor cell line (Fig. S2A) – with less sensitivity in the xenobiotic metabolically-active B-13/H hepatocyte cell derived therefrom (Fig. S2D). Since there was a lack of apparent sensitivity in H69 (Fig. S2G) and MCF-7 cells (Fig. S5A), the nature of this toxicity was examined in the B-13 liver progenitor cell. The earliest observed effects of PBS 1 and 2 extracts on B-13 cells was an inhibition in proliferation, which was confirmed by marked inhibition of DNA synthesis (Fig. 2A), initial inhibition in cell numbers, and subsequent cell death between 24 h and 48 h after exposure (Fig. 2B). A dose-response effect of the toxic PBS extracts on B-13 MTT reduction is demonstrated (Fig. 2C). Since the cells excluded trypan blue after effects on proliferation and MTT reduction, it was hypothesized that B-13 cells undergo an apoptotic mechanism of cell death. PBS extracts 1 and 2 induced a marked increase in caspase 3/7 activity (Fig. 2D) and cleavage of genomic DNA to generate classic nucleosomal ladders (Fig. 2E), supporting an initial apoptotic mode of cell death.

**Fig. 3:**
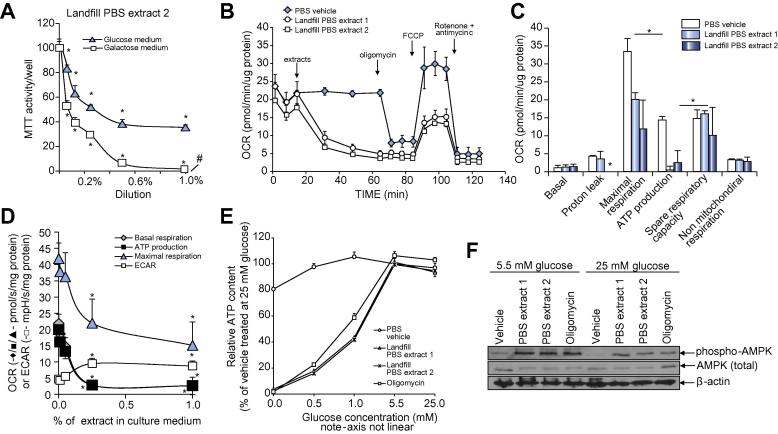
**Landfill site PBS extracts inhibit mitochondrial oxidative phosphorylation in B-13 cells.** (A) B-13 cells cultured in normal media (containing 5.5 mM glucose) or in media with the glucose substituted for 5.5 mM galactose for at least two weeks prior to exposure to the indicated landfill PBS extract 2 dilution for 24 h, followed by MTT reduction activity determination. Data are expressed relative to vehicle treated cells and are the mean and SD of three separate treatments from the same experiment typical of at least three separate experiments. Similar results were obtained with landfill PBS extract 1. *Significantly different from 0%/PBS solvent control (*p* < 0.05) using ANOVA/Bonferroni-Holm comparison between groups. (B) Time course of OCR in B-13 cells treated with the indicated landfill extract. OCR was determined using a Seahorse XF analyzer with the injections of 1% (v/v) PBS extracts, 1 µM oligomycin, 1 µM FCCP, 0.5 µM and 0.5 µM rotenone and antimycin A, respectively, as indicated. Readings were normalized to protein concentration and are the mean and SD of at least four readings from the same experiment, typical of at least three separate experiments. (C) Effect of 1% PBS extracts on the B-13 mitochondrial functions based on seahorse time-course data. Data are the mean and SD of at least four readings from the same experiment, typical of at least three separate experiments. *Significantly different from PBS solvent control (*p* < 0.05) using ANOVA/Bonferroni-Holm comparison between groups. (D) Dose-response effects of PBS extracts on B-13 mitochondrial function and ECAR. Data are the mean and SD of at least four readings from the same experiment, typical of at least three separate experiments. *Significantly different from 0%/PBS solvent control (*p <* 0.05) using ANOVA/Bonferroni-Holm comparison between groups. (E) B-13 cells were treated with environmental samples at 1% (v/v) or 1 µM oligomycin for 2 h at the indicated glucose concentration prior to determination of ATP content. Data are expressed relative to vehicle treated cells at 5.5 mM glucose and are the mean and SD of three experiments, typical of at least three separate experiments. (F) B-13 cells were treated with the indicated environmental samples at 1% (v/v) or 1 µM oligomycin in culture medium containing the indicated concentration of glucose for 24 h prior to Western blot analyses for phosphor-AMPK (AMPK phosphorylated at residue Thr172), total AMPK and β-actin. ECAR, extracellular acidification rate; OCR, oxygen consumption rate; PBS, phosphate buffered saline.

To further characterize the causative mechanism(s) inducing B-13 cell apoptosis, the potential role of mitochondria was examined, since this organelle plays a critical role in apoptotic mechanisms of cell death. Replacing media glucose with galactose has been reported to increase cellular susceptibility to mitochondrial toxicants.^24^ It was demonstrated that replacing glucose with galactose sensitized B-13 cells – in a dose responsive manner – to the toxic effects of the toxic PBS extracts (Fig. 3A). Measurements of oxygen consumption rates in B-13 cells using a seahorse analyzer confirm that the toxic PBS extracts inhibited oxidative phosphorylation, targeting in particular maximal respiration and ATP production (Fig. 3B, C). B-13/H mitochondria were less sensitive to the toxic PBS extracts, experiencing weaker inhibitory effects on both maximal respiration rate and ATP production (see Fig. S8A), supporting a role for the mitochondrial effects of toxic PBS extracts in B-13 cell death. Toxic PBS extract exposure resulted in a dose-dependent decrease in oxygen consumption rate (OCR) and a dose-dependent increase in extracellular acidification rate (ECAR) in B-13 cells (Fig. 3D). The increase in ECAR was considered likely due to an inhibition of mitochondrial oxidative phosphorylation and compensatory regulated changes in metabolism such as glycolytic flux. This hypothesis was supported by decreases in cellular ATP in cells treated with toxic PBS extracts when glucose was limited (Fig. 3E); a protection from toxicity through increasing glucose concentration (data not included) and an activation of AMP activated protein kinase by phosphorylation, an effect not observed when cells were treated in high glucose media (Fig. 3F).

These data indicate that the PBS extracts from two sampling sites in close proximity to the landfill site contain a chemical(s) that inhibited mitochondrial oxidative phosphorylation.

### The landfill toxic PBS extracts inhibit cultured human cholangiocyte mitochondrial function

**Fig. 4:**
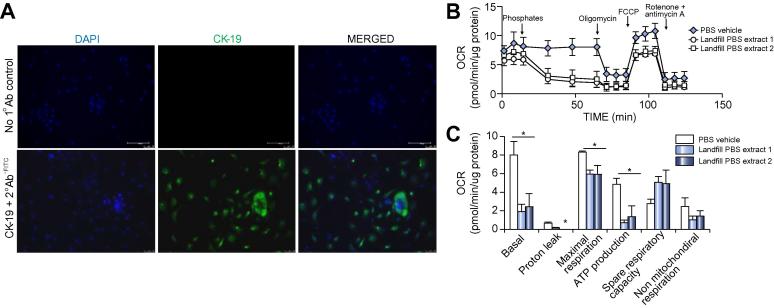
**Landfill site PBS extracts inhibit mitochondrial oxidative phosphorylation in primary human cholangiocytes.** (A) immunocytochemistry for the cholangiocyte marker CK-19. DAPI was used to identify cell nuclei. (B) Time-course plot of OCR in primary cultures of human cholangiocytes treated with the indicated landfill extract. OCR was determined using a Seahorse XF analyzer with the injections of 1% (v/v) PBS extracts, 1 µM oligomycin, 1.5 µM FCCP, 0.5 µM and 0.5 µM rotenone and antimycin A, respectively, as indicated. Readings were normalized to protein concentration and are the mean and SD of at least four separate determinations with cells from the same donor, typical of results from cells isolated from three donors. (C) effect of 1% PBS extracts on the human cholangiocyte mitochondrial functions based on seahorse time-course data. Data are the mean and SD of at least four readings from the same experiment, typical of results from three donors. *Significantly different from PBS solvent control (*p* < 0.05) using ANOVA/Bonferroni-Holm comparison between groups. CK-19, cytokeratin 19; OCR, oxygen consumption rate; PBS, phosphate buffered saline. (This figure appears in colour on the web.)

To establish whether the toxic response of B-13 cells to toxic PBS extracts could be relevant to man, cholangiocytes were isolated from resected human liver and exposed to toxic PBS extracts or control PBS, although limitations in tissue and cell numbers precluded extensive analyses. The cells isolated from three donors expressed the biliary marker cytokeratin 19, confirming that an enriched population of cholangiocytes was isolated and cultured (Fig. 4A). Application of the seahorse approach to determine effects on OCR demonstrated that both toxic PBS extracts inhibited mitochondrial function in a similar manner to that observed in B-13 cells (Fig. 4B). Maximal respiration and ATP production were also affected by the PBS extracts, as observed with B-13 cells, additionally, basal respiration rates were also inhibited (Fig. 4C).

Therefore, these limited analyses in human cholangiocytes suggest that the adverse effects of the toxic PBS extracts could be relevant in man if the ductular regions of the liver are exposed to sufficient concentrations of the chemical(s).

### The toxic chemical present in aqueous soil extracts from near landfill was the ionic liquid 3-methyl-1-octyl-1H-imidazol-3-ium^+^

**Fig. 5:**
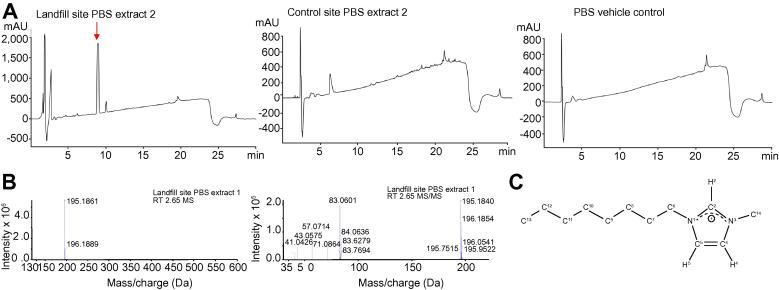
**The chemical primarily responsible for the toxic effects associated with landfill PBS extracts 1 and 2 is M8OI.** (A) HPLC chromatograms of PBS extracts from the indicated sites with the peak associated with toxicity indicated (by arrow, top panel), toxicity data not included. These data are from pooled fractions collected by preparative HPLC at the retention time associated with toxicity in landfill site PBS extract 2, and therefore partially-purified from soil PBS extracts on the basis of partition into the aqueous PBS phase and preparative HPLC. Control PBS extract 2 (middle panel) is provided as a chromatogram from a PBS extract not displaying any toxic effects in B-13 cells and was typical of other non-toxic PBS extracts from both control and landfill sites. (B) Mass spectrometry (upper panel) and tandem mass spectrometry (lower panel) for peak in panel A associated with toxicity in B-13 cells. (C) Predicted structure of peak in panel A associated with toxicity in B-13 cells, based additionally on NMR data (see Fig. S7A,B). HPLC, high performance liquid chromatography; NMR, nuclear magnetic resonance; PBS, phosphate buffered saline. (This figure appears in colour on the web.)

**Fig. 6:**
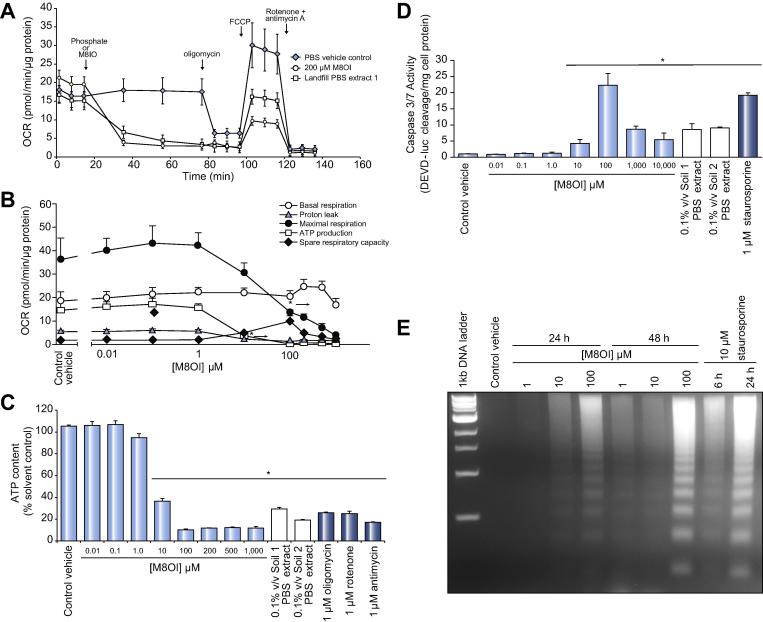
**M8OI recapitulates the mitochondrial effects and induces apoptosis in B-13 cells.** (A) Time course of OCR in B-13 cells treated with the indicated landfill extract. OCR was determined using a Seahorse XF analyzer with the injections of 1% (v/v) PBS extracts, 1 µM oligomycin, 1 µM FCCP, 0.5 µM and 0.5 µM rotenone and antimycin A respectively as indicated. Readings were normalized to protein concentration and are the mean and SD of at least four readings from the same experiment, typical of at least three separate experiments. (B) Dose-response effects of M8OI on mitochondrial parameters in B-13 cells, *Significantly different OCR from control vehicle at this concentration of M8OI and higher (*p* < 0.05) using ANOVA/Bonferroni-Holm comparison between groups. (C) Effect of authentic M80I (chloride salt), PBS extracts or other indicated mitochondrial toxins on ATP content in B-13 cells after 2 h exposure. Data are the mean and SD of three separate treatments for the same experiment typical of at least three separate experiments. ^*^Significantly different from PBS control vehicle (*p* < 0.05) using ANOVA/Bonferroni-Holm comparison between groups. (D) Effect of authentic M80I (chloride salt), PBS extracts or staurosporine on caspase 3/7 activities in B-13 cells after exposure for 2 days. Data are the mean and SD of three separate treatments for the same experiment typical of at least three separate experiments. *Significantly different from PBS control vehicle (*p <* 0.05) using ANOVA/Bonferroni-Holm comparison between groups. (E) B-13 cells were treated with the indicated concentration of M8OI or staurosporine for the indicated periods prior to genomic DNA isolation and analysis for nucleosomal ladder formation. OCR, oxygen consumption rate; PBS, phosphate buffered saline.

PBS extracts from landfill sampling sites 1 and 2, were subjected to thin-layer chromatography and spectrophotometry and determined to be a non-fluorescent chemical(s) lacking extensive bond conjugation (Fig. S6A–C). The presence of bacterial agents and/or toxic proteins was also excluded (Fig. S6D, E). Exposing B-13 cells to separate HPLC fractions showed that the toxic effects associated with the extracts co-eluted with the major peak detected, which was not detectable in the control and non-toxic landfill PBS extracts after identical batch fraction collection (Fig. 5A). Mass spectrometric analyses of the material eluting with this peak suggested the presence of a single species corresponding to a singly charged [M+H] of 195.1861 Da (Fig. 5B). Interrogation of databases predicted the best elemental fit to the parent ion of C_12_H_22_N_2_, with a mass error of 5 ppm. The isotope pattern did not suggest the presence of chlorine or sulfur in the molecule. Mass spectrometry and fragmentation analyses suggested that the chemical consists of two alkyl chains and a cyclic moiety containing the two nitrogen atoms (Fig. 5B). However, the predicted molecular formula could, in theory, be generated by 3,316 potential distinct chemical structures according to Chemspider (http://www.chemspider.com/). Therefore, repeated fractions containing this peak were collected, pooled and subjected to NMR analysis (Fig. S7), identifying the chemical as the ionic solvent 3-methyl-1-octyl-1H-imidazol-3-ium^+^ (M8OI), see Fig. 5C for structure. Exposing B-13 cells to the authentic commercially-available chloride salt (M8OI-Cl) essentially replicated the toxicity of PBS 1 and 2 extracts in terms of effects on mitochondrial function (Fig. 6A) including a clear dose-response effect on maximal respiration and ATP production (Fig. 6B), ATP levels (Fig. 6C), caspase 3/7 activities (Fig. 6D), MTT reduction (Fig. S10A) and induction of DNA nucleosomal laddering (Fig. 6E).

An approximate EC_50%_ for MTT of 0.2% (v/v) for landfill PBS extract 2 (Fig. 2C) and 50 µM M8OI for B-13 cells (Fig. S8B), suggests that the concentration of M8OI in the PBS extract 2 was approximately 25 mM.

B-13 cells also showed an increased sensitivity to M8OI compared to the epithelial B-13/H cell in terms of mitochondrial sensitivity (Fig. S8A), caspase 3/7 induction and loss of MTT reduction activity (Fig. S8B), and the minimum M8OI concentration induced a detectable DNA ladder (Fig. S8C). M8OI was mildly toxic to the human cholangiocyte-like H69 cell line (Fig. S9) based on MTT reduction assays (EC_50%_ ∼500 µM compared to ∼100 µM for B-13 cells).

These data indicate that soil samples surrounding a landfill waste site contained the ionic liquid M8OI and that this chemical is toxic to liver progenitor cells through an interaction with their mitochondria, resulting in induction of apoptosis.

### M8OI is metabolized to a carboxylate-containing metabolite by human hepatocytes and is capable of being enzymatically incorporated into the E2 component of pyruvate dehydrogenase

**Fig. 7:**
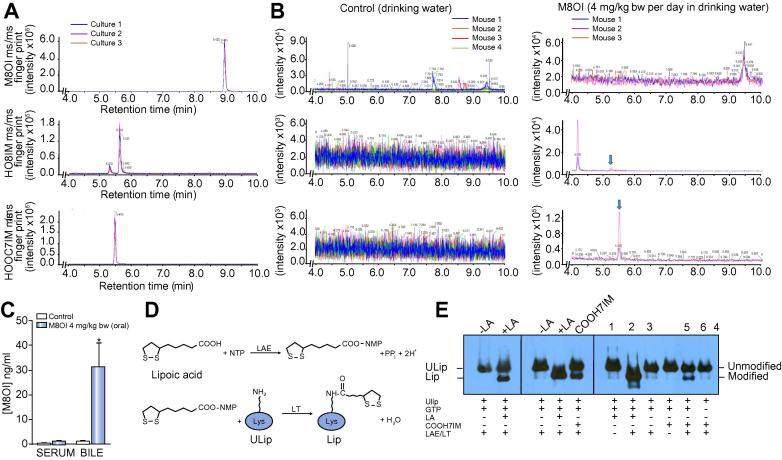
**M8OI is metabolized to COOH7IM in human hepatocytes and mice and is enzymatically incorporated into an unlipoylated fragment of PDC-E2 *in vitro*.** (A) Liquid chromatography-high resolution tandem mass spectrometry, using a TripleTOF 5600 high-resolution quadrupole time-of-flight mass spectrometer (Sciex), analyses of three separate human hepatocyte cultures incubated with M8OI for 24 h prior to detection of M8OI, HO8IM and COOH7IM. (B) Detection of M8OI in mouse sera orally exposed to control (left panels) or M8OI (right panels) in their drinking water as outlined in the methods section. Position of HO8IM and COOH7IM peaks is indicated by arrows. (C) Mean and standard deviation concentration of M8OI in mouse sera and gall bladder bile in control (three mice) and M8OI exposed mice (five mice). *Significantly different from control bile (*p* < 0.05) using the Student’s *t* test (two tailed). (D) Illustration of two-step procedure for incorporation of LA into a ULip. (E) Western blot for the detection of ULip and Lip after addition of LA or COOH7IM, with additions as indicated. LA, lipoic acid; LAE, lipoate activating enzyme; LT, lipoyl-AMP(GMP):N-lysine lipoyl transferase; ULip, unlipoylated fragment of PDC-E2; Lip, lipoylated fragment of PDC-E2. (This figure appears in colour on the web.)

Lipoic acid (structure in Fig. 7D) is a small endogenously synthesized co-factor that is covalently bound to a variety of enzymes, including the PDC-E2. This co-factor is essential for the acyl or methylamine transfer functions of the enzymes to which it is linked.[10], [11] Lipoic acid may be synthesized *de novo* from octanoic acid produced during fatty acid synthesis in bacteria. There is also a scavenging pathway that links ATP or GTP hydrolysis to its activation and conjugation to proteins^25^ (Fig. 7D).

Although M8OI is persistent it has been shown to be broken down in the environment through oxidation of the terminal carbon (C_13_) to form the carboxylic acid – 1-(7-carboxyheptyl)-3-methyl-1H-imidazol-3-ium (COOH7IM).^26^ This pathway of catabolism by soil bacteria mirrors the pathway of fatty acid omega oxidation observed in mammalian liver (see Fig. S10B). COOH7IM was not acutely toxic to B-13 cells (data not shown) and therefore it was not directly tracked using our approach. Therefore, its presence or absence in soil samples cannot be determined with certainty.

To determine whether M8OI is metabolized to COOH7IM, cultured human hepatocytes were exposed to M8OI for 24 h prior to analysis by TripleTOF 5600 high-resolution quadrupole time-of-flight mass spectrometry (Sciex). Mass spectrometric scanning of predicted masses for the parent M8OI, hydroxylated metabolite (HO8IM) and COOH7IM was used for detection and to demonstrate that M8OI was metabolized to HO8IM and predominantly COOH7IM as predicted (Fig. 7A). Analysis of serum samples isolated from mice orally administered M8OI demonstrated that M8OI was absorbed from the gastrointestinal tract, was systemically available and metabolized in the mouse like in man, since both HO8IM and COOH7IM were detectable in sera (Fig. 7B). Interestingly, M8OI was found to be present in bile at levels >30-fold the concentrations found in sera (Fig. 7C), indicating that a proportion of the chemical is cleared intact via biliary excretion. COOH7IM was synthesized and its enzymatic incorporation into PDC-E2 examined. The covalent incorporation of lipoic acid into an unlipoylated fragment of the human PDC-E2 protein (ULip) is shown (Fig. 7E), as previously described.^27^ The incorporation of the M8OI derivative possessing a carboxylated group on C_13_ (center panel, COOH7IM) was also shown (Fig. 7E), an effect not observed with M8OI itself nor an M8OI derivative possessing a hydroxyl group on C_13_ (*i.e.* HO8IM, data not shown). COOH7IM incorporation into ULip was both GTP- and enzyme-dependent (Fig. 7E, right panel).

## Discussion

This study initially aimed to determine the feasibility of extracting chemicals from soil samples and applying them to a selected suite of *in vitro* toxicity assays in order to screen for any toxicological effects. The hypothesis was that the approach would detect higher levels of chemicals with potential toxic effects around a landfill site compared to sample sites not in proximity to a landfill site. The presence of chemicals activating the AHR was examined because this receptor is known to be activated by PAHs associated with fossil fuel burning and industrial pollution.^28^ This receptor mediates most of the toxic effects of dioxin in animals and man, including chloracne, anorexia, thymic atrophy and cancer.^29^ The AHR has also been shown to recognize both xenobiotics and natural compounds (tryptophan metabolites, dietary components and microbiota-derived factors), and is important for maintenance of homeostasis at mucosal surfaces through regulation of type 3 innate lymphoid cells and T helper 17 cell activities.^30^ The PPARα is the nuclear receptor drug target for fibrate drugs that mediates reductions in lipid levels through increasing peroxisomal oxidation of fatty acids.^31^ The presence of chemicals activating the PPARα was examined because environmental surfactant pollutants (*e.g.* perfluoroalkyl acids) activate this receptor.^18^ However, PPARα agonists have also been shown to be protective against cholestatic liver injury^32^ and are currently being considered as therapies for PBC and other cholestatic liver diseases.^33^ One conclusion from this study is that it is possible to readily detect chemicals from soil extracts that contact and activate the receptors. The results of our analyses indicate that the chemicals activating the AHR or PPARα were widely present in the soils (either naturally and/or through anthropomorphic activity), including soils not in close proximity to a landfill site. Comparison of the potentially toxic element content of these soils, based on metals and polyaromatic hydrocarbon (PAH) analyses, indicates that the soil samples are consistent with soils from urban areas in north east England, and in most cases are lower than the mean for soils in this region.^34^

In contrast, chemicals that activated the ERα were at higher levels in soils around a landfill site compared to control soils. A wide variety of man-made chemicals have been suggested to have endocrine-disrupting chemicals in animals and humans[35], [36] and many have been shown to have estrogenic properties in that they mimic the biological effects of endogenous estrogens (xenoestrogens). Therefore, in terms of disposal of normal domestic waste, there may be a potential for xenoestrogens to be at higher levels in the surrounding environment, although whether this is hazardous to the environment and to nearby populations remains to be determined.

Given the marked toxic effect of two PBS extracts from two sites in close proximity to a landfill site, the mechanism of action and identity(ies) of the chemical(s) responsible were investigated. Less potent effects were observed in B-13/H hepatocytes derived from the B-13 progenitor (perhaps due to degradation to less toxic metabolites in these more active xenobiotic metabolizing cells) and a variety of other cells such as human cholangiocytes. A single chemical was identified – the ionic liquid 3-methyl-1-octyl-1H-imidazol-3-ium^+^ (M8OI) in the toxic PBS extracts (which also contained estrogenic activity). Since the pure commercially-available chemical induced the same effects in B-13 cells, it is likely that the M8OI alone was responsible for the effects of the toxic PBS extracts. Retrospective non-targeted data independent liquid chromatography-high resolution tandem mass spectrometry analysis of all PBS extracts confirmed that landfill sites 1 and 2 contained high levels of M8OI (the highest – PBS extract 2, 2.4 mg/ml equal to 12 mM) in good agreement with the estimation from toxicity data of ∼25 mM. M8OI was present at low levels in all other landfill sampling PBS extracts (range 0.0012–0.038 mg/L) but not detected in any control sample sites. Based on these analyses, and assuming that all M8OI partitioned into the PBS extract, this means the highest M8OI soil concentration was 0.48 mmole/kg (equivalent to 94 mg/kg soil).

Ionic liquids are salts with a melting temperature below the boiling point of water, are often liquids at room temperature and are often composed of organic cations and inorganic anions.^37^ They have been proposed to be a new generation of “green solvents” because they are organic solvents with relatively low volatility. Therefore, they have many of the advantages of molten salts without requiring high temperatures.^37^ The first ionic liquid was identified in the mid-19th century, but the modern era of discovery and development occurred with the generation of 1-butylpyridinium chloride–aluminium chloride mixtures in the 1970s.[38], [37] They may currently find use in protection products and herbicidal ionic liquids such as derivatives of phenoxyacids exhibiting a selective herbicidal activity against dicotyledonous plants.^39^ According to commercial suppliers, M8OI is used/associated with metal plating, electropolishing, metal reprocessing, phase transfer media, batteries, nanomaterials, industrial solvents, nuclear fuel red waste, enzymatic catalysis, lubricants, heat transfer and solar energy conversion. Interrogation of the ECHA database shows that five different M8OI salts have been pre-registered, but since no data on their toxicity have been submitted, their production level by any single producer should be less than 100 tonnes/annum (Dr D Bell, ECHA, personal communication). However, it should be noted that M8OI is one of many structurally-related ionic liquids, some of which are used more widely.

A limited number of studies have been completed with salts of M8OI, with only one study available to our knowledge in a mammalian species *in vivo*. In this respect, the acute toxic effects of 1-methyl-3-octylimidazolium bromide [M8IO+Br−] have been examined in mice. The study is limited to potential adverse effects up to 24 h after i.p. administration with the authors calculating an LD_50%_ of 35.7 mg/kg body weight.^40^ The authors report histopathological changes in the liver 10 h after administration.

PBC is a chronic cholestatic liver disease characterized by clinical chemical markers of periportal injury (*e.g.* raised serum ALP), histopathological changes in the periportal region (intrahepatic bile duct loss; mixed phenotype periportal inflammation and fibrosis) and high serum titers of antibodies to antigens present on the inner mitochondrial membrane (AMA).[10], [11] AMA is directed to the 2-oxoacid dehydrogenases complex family of enzymes in over 95% of patients with PBC, likely through loss of tolerance to the dihydrolipoamide acetyltransferase (E2) and/or enzyme 3 binding protein components of the pyruvate dehydrogenase complex (*i.e.* PDC-E2 and PDC-E3BP respectively).[41], [10], [11] There is likely a genetic link to likelihood of developing PBC since there is a sibling relative-risk of approx. 10.^42^ A number of polymorphisms have been associated with disease risk such as HLA-DR8, with an odds ratio ranging from 2.4 to 3.3 depending on the population examined.^4^ A number of other loci have been identified by genome-wide association studies as influencing disease risk, suggesting involvement of the innate and adaptive immune systems and signaling via the NF-κB, toll-like receptor and tumor necrosis factor pathways in PBC etiology.^5^ However, there remains an environmental element to disease prevalence (which might account in part for an apparent genetic association to disease incidence because most families inhabit the same environment). In this respect, epidemiological studies over the last few decades have suggested a link between PBC incidence and the reservoir source of drinking water;^6^ areas of heavy mining;^7^ proximity to toxic landfill sites^8^ and exposure to chemicals, such as those used in hair dyes.^9^ These observations have been accompanied by a number of molecular studies which lend weight to the potential for xenobiotics to be a trigger for PBC. The first of these was the observation that the synthesis of peptides in which the lipoate moiety had been replaced with xenobiotic lipoate mimics showed immunoreactivity to PBC patient sera.^43^ Rabbits immunized with 6-bromohexanoate coupled to bovine serum albumin (BSA), but not BSA-immunized controls, developed AMA that was capable of inhibiting PDC-E2 enzymatic function and binding to peptide sequences not present in the xenobiotic carrier immunogen.^44^ 2-Octynoic acid – used in cosmetics – was first identified as a potential xenobiotic trigger through screening 107 potential xenobiotic mimics coupled to the lysine residue of the immunodominant 15 amino acid peptide of the PDC-E2 inner lipoyl domain. PBC patient sera demonstrated high Ig reactivity against 2-octynoic acid-PDC-E2 peptide.^45^ Optimal chemical structure of the xenobiotically modified epitope recognized by AMA-positive PBC sera was subsequently determined to be 2-nonynoic acid.^46^ However, immunizing C57BL/6 mice with 2-octynoic acid coupled to BSA resulted in autoimmune cholangitis; increased serum AMA; increased liver lymphoid cell numbers; increases in CD8(+) liver infiltrating cells, particularly CD8(+) T cells that co-express CD44 and an elevation in serum tumor necrosis factor-alpha and interferon-gamma levels.^47^ Similar effects were also seen in a non-obese diabetic (NOD) congenic strain.^48^ At the same time, it was demonstrated that recombinant lipoylation enzymes were capable of aberrantly incorporating xenobiotic lipoic acid analogues – including octanoic, hexanoic acids and the xenobiotic 6 bromohexanoic acid – into PDC-E2.^27^ More recent studies have shown that AMA-positive PBC sera demonstrate reactivity to xenobiotics that have lipoic acid covalently modified at the disulfide ring, therefore without replacing the lipoic acid.^49^ In both early and late-stage PBC the predominant Ig isotype to 6,8-bis(acetylthio) octanoic acid (SAc)-conjugated BSA was IgM, with titers higher with advanced stage disease.^50^ However, whilst these studies show that exposure to a haptenized xenobiotic can give rise to immunological and pathological changes in animal models similar to the PBC in humans, none of the studies have exposed animals to xenobiotic alone to produce these effects.

It can be seen that M8OI bears some structural similarity to lipoic acid and, if metabolized to a carboxylic acid (in the environment and/or in the liver), has the potential to be incorporated into PDC-E2 in place of lipoic acid (Fig. S10B). It could be envisaged that M8OI undergoes omega hydroxylation, which would occur via hepatic cytochrome P450 hydroxylation of fatty acids, followed by oxidation of the alcohol to a carboxylic acid.^51^ An additional factor that is likely to be important in disease pathogenesis is the biliary route of excretion of these chemicals. Preliminary data in this manuscript indicate that a proportion of the intact toxic M8OI chemical passes into the bile and that concentrations may be significantly higher than those present in serum.

This paper identifies, for the first time, a xenobiotic present in the environment capable of being a potential xenobiotic trigger for PBC. It remains to be determined whether M8OI is a significant risk for triggering PBC, given its low production levels. However, since M8OI is structurally-related to other more widely used ionic liquids, there may be a hazard with these chemicals relating to PBC. However, the suggestion that structurally-related ionic liquids represent a risk for triggering PBC remains hypothetical. A realistic assessment of risk may be determined through exposing experimental animals more extensively to these ionic liquids.

## Financial support

This work was funded by a grant from the Institute of Sustainability (Newcastle University) and was in part, funded by the National Institute for Health Research Health Protection Research Unit (NIHR HPRU) in Chemical and Radiation Threats and Hazards in partnership with Public Health England (PHE), the MRC (in the form of an ITTP studentship supporting A.C.L.), the Newton-Mosharafa Fund (in the form of a studentship supporting T.M.A) and by a research grant from the LIVErNORTH charity (to P.M.P and M.C.W.). NIHR HPRU funding was used for the part of the research that did not involve any live animals. The views expressed are those of the authors and not necessarily those of the NHS, the NIHR, the Department of Health or PHE.

## Conflict of interest

The authors declare they have no conflicts of interests. The views expressed are those of the authors and not necessarily those of the NHS, the NIHR, the Department of Health or Public Health England.

Please refer to the accompanying ICMJE disclosure forms for further details.

## Authors’ contributions

P.M.P., A.C.L., M.P.D and S.K.M. performed the majority of the laboratory-based work and analyses presented in the manuscript. J.M.P., A.K.L., T.M.A., M.P.C., H.T., L.I.B., A.K.R anf R.F. performed a portion of the laboratory experiments and their related analyses. C.W. and W. McF. Performed and analyzed NMR data. A.O., C.W., G.E.N., D.E.J. and P.G.B, provided advice and contributed to the experimental design. M.C.W conceived the studies, designed the experiments and wrote the manuscript. All authors read and commented on the final manuscript.
