# Supplementary material for: Identification of a xenobiotic as a potential environmental trigger in primary biliary cholangitis
Source: J Hepatol. 2018 Nov;69(5):1123–35. doi: 10.1016/j.jhep.2018.06.027 (PMC6192827; doi:10.1016/j.jhep.2018.06.027)
Supplement: Supplementary data [file CTAT_table.pdf]

EASL | JOURNAL OF HEPATOLOGY

Journal of Hepatology

CTAT methods

Tables for a "Complete, Transparent, Accurate and Timely account" (CTAT) are now mandatory for all revised submissions. The aim is to enhance the reproducibility of methods.

- Only include the parts relevant to your study
- Refer to the CTAT in the main text as 'Supplementary CTAT Table'
- Do not add subheadings
- Add as many rows as needed to include all information
- Only include one item per row

If the CTAT form is not relevant to your study, please outline the reasons why:

1.1 Antibodies

Name	Citation	Supplier	Cat no.	Clone no.
Anti-Cyp1a1		Daiichi Chemical Co (Tokyo)	n/a	n/a
Anti-phospho-AMPK		Cell Signalling Technologies	2535S	40H9
Anti-AMPK		Cell Signalling Technologies	2603S	23A3
Anti-β-actin		Sigma Chemical Co	A1978	AC-15
Anti-CK-19		Abcam	ab84632	n/a
Anti-AMA	Walden HR et al. Xenobiotic incorporation into pyruvate dehydrogenase complex can occur via the exogenous lipoylation pathway. Hepatology. 2008;48:1874-84.	Not commercially available – obtained from PBC patients.	-	-

n/a = not available

1.2 Cell lines

Name	Citation	Supplier	Cat no.	Passage no.	Authentication test method
B-13	Mashima H et al. Formation of insulin-producing cells from pancreatic acinar AR42J cells by hepatocyte growth factor. Endocrinology. 1996;137(9):3969-76.	Not commercially available – originally provided by Prof David Tosh (Bath University, UK).	n/a	>50	Confirmed cytogenetics (Fairhall EA et al. The B-13 hepatocyte progenitor cell resists pluripotency induction and differentiation to non-hepatocyte cells. Toxicology Research 2, 308 -

					320 (2013). Species (rat) confirmed by PCR-RLFP analysis of the mitochondrial genome-encoded cytochrome b according to Losi et al. (2008) using primers L14816 and H15173 for species determination.
H69	Grubman SA et al. Regulation of intracellular pH by immortalized human intrahepatic biliary epithelial cell lines. Am J Physiol. 1994;266:G1060-70.	Not commercially available – originally obtained from Prof J Kirby (Newcastle University, UK).	n/a	>50	n/a.
MCF7	Soule HD et al. A human cell line from a pleural effusion derived from a breast carcinoma. J Natl Cancer Inst. 1973;51:1409-16.	European collection of authenticated cell lines (ECACC) - Public Health England).	86012803	>50	See ECACC
HepG2	Knowles BB et al. Human hepatocellular carcinoma cell lines secrete the major plasma proteins and hepatitis B surface antigen. Science. 1980;209:497-9.	European collection of authenticated cell lines (ECACC) - Public Health England).	85011430	>50	See ECACC
AR-EcoScreen™	OECD guideline 458 (2016).<br><br>Ohyama SA et al. Study on anti-androgenic effects of bisphenol a diglycidyl ether (BADGE), bisphenol F diglycidyl ether (BFDGE) and their derivatives using cells stably transfected with human androgen receptor, AR-EcoScreen. Food Chem Toxicol. 2004 Jun;42:983-93.	JCRB Cell Bank (Osaka, Japan), National Institute Biomedical Innovation, health and Nutrition.	JCRB1328	>3	See JCRB Cell Bank.

1.3 Organisms

Name	Citation	Supplier	Strain	Sex	Age	Overall n number
E. *coli*		ThermoFisher, Paisley, UK	TOP10	n/a	n/a	n/a
Mice	Wallace K et al. The PXR is a drug target for chronic inflammatory liver disease. J Steroid Biochem Mol Biol. 2010; 120: 137-48.	Bred in house	C57Bl6 mice bearing a transgene composed of three NF-κB sites from the Ig κlight chain promoter coupled to the gene encoding firefly luciferase	Female	15-16 months	11

1.4 Sequence based reagents

Name	Sequence	Supplier

1.5 Biological samples

Description	Source	Identifier

1.6 Deposited data

Name of repository	Identifier	Link

1.7 Software

Software name	Manufacturer	Version

1.8 Other (e.g. drugs, proteins, vectors etc.)

1.9 Please provide the details of the corresponding methods author for the manuscript:

**Professor Matthew Wright**

2.0 Please confirm for randomised controlled trials all versions of the clinical protocol are included in the submission. These will be published online as supplementary information.

