# Supplementary material for: Identification of a xenobiotic as a potential environmental trigger in primary biliary cholangitis
Source: J Hepatol. 2018 Nov;69(5):1123–35. doi: 10.1016/j.jhep.2018.06.027 (PMC6192827; doi:10.1016/j.jhep.2018.06.027)
Supplement: Supplementary data 1 [file mmc1.pdf]

Identification of a xenobiotic as a potential environmental trigger in primary biliary cholangitis

Philip M. Probert, Alistair C. Leitch, Michael P. Dunn, Stephanie K. Meyer, Jeremy M. Palmer, Tarek M. Abdelghany, Anne F. Lakey, Martin P. Cooke, Helen Talbot, Corinne Wills, William McFarlane, Lynsay I. Blake, Anna K. Rosenmai, Agneta Oskarsson, Rodrigo Figueiredo, Colin Wilson, George E. Kass, David E. Jones, Peter G. Blain, Matthew C. Wright

Table of contents

Supplementary materials and methods…………………………………………………2

Fig. S1……………………………………………………………………………………..14

Fig. S2……………………………………………………………………………………..16

Fig. S3……………………………………………………………………………………..18

Fig. S4……………………………………………………………………………………..20

Fig. S5……………………………………………………………………………………..22

Fig. S6……………………………………………………………………………………..24

Fig. S7……………………………………………………………………………………..26

Fig. S8……………………………………………………………………………………..32

Fig. S9……………………………………………………………………………………..34

Fig. S10……………………………………………………………………………………35

Fig. S11……………………………………………………………………………………37

Table S1…………………………………………………………………………………..38

Table S2…………………………………………………………………………………..39

Table S3…………………………………………………………………………………..40

References………………………………………………………………………………..41

Supplementary materials and methods

Chemicals

3-methyl-1-octyl-1H-imidazol-3-ium **(**M8OI) was purchased from Sigma (Poole, UK). 1-(8-hydroxyoctyl)-3-methyl-imidazolium (HO8IM) and 1-(7-carboxyheptyl)-3-methyl-1H-imidazol-3-ium (COOH7IM) were custom synthesized with purity and chemical structures determined by HPLC, mass spectrometry and NMR techniques (for COOH7IM, see **Supplementary Fig. 11**).

Preparation of soil extracts

Surface soil samples (0-5cm in depth) were collected and extraneous vegetable matter and stones removed. Each sample was divided into four 250g portions. A sample of one portion was digested using *aqua regia* in accordance with BS7755 for metals analysis. Two portions were subjected to either methanol (for polar molecule) or chloroform (for hydrophobic chemical) extractions by sonicating with 300mls of solvent for 10 mins, followed by addition of a further 100mls of solvent and sonication for a further 10 mins prior to filtration with 25µm filters and collection of filtrate. Filtrates were evaporated in a rotary evaporator and then blown down to near dryness under a stream of nitrogen. The methanol extracted material was divided into two and added to either 10mls of phosphate buffered saline (PBS, 137 mM NaCl, 27 mM KCl, 100 mM phosphate pH 7.4) or 10mls ethanol. The chloroform extracted material was re-dissolved into 10mls chloroform. The solvated extracted chemicals were then separated from any precipitate and stored at -20°C (ethanol and chloroform extracts) or 4°C (PBS extracts).

Thirteen soil samples were collected from allotments, footpaths and the roadside verges surrounding an urban landfill site. Three control soil samples were collected from 3 separate sites. One sample was obtained from the University farm in rural Northumberland at a site with controlled fertiliser regime for the last 130 years. The remaining 2 control samples were obtained from gardens in urban areas in the region.

Metal analysis

Metals (As, B, Ba, Cd, Co, Cr, Cu, Pb, Mn, Hg, Mo, Ni, Se and Zn) were determined in soil directly via *aqua regia* digestion. Metals were also analysed in the methanol extraction after solubilisation with 10mls of 1% nitric acid and were at, or below, the limit of detection (< 0.1mg/L). Metal concentrations were determined using a Varian Vista MPX Inductively Coupled Plasma Optical Emission Spectrometer (ICP-OES), with five calibration standards ranging from 1-100 mg/L in accordance with BS ISO 22036:2008.

PAH analysis

The chloroform extracts were analysed for PAHs by GC-MS analysis using an Agilent 6890/7890A GC in split less mode (injector at 280°C) linked to a Agilent 5975C MSD (electron voltage 70eV, source temperature 230°C, quad temperature 150°C multiplier voltage 1800V, interface temperature 310°C). The acquisition was controlled by an HP Compaq computer using Chemstation software, initially in full scan mode (50-600 amu/sec) or in selected ion mode (30 ions, 0.7cps, 35ms dwell) for greater sensitivity. The sample (1ul), in hexane, was injected by an HP7683B auto sampler and the split opened after 1 minute. After the solvent peak had passed the GC temperature programme and data acquisition commenced. Separation was performed on a Phenomonex fused silica capillary column (30m x 0.25mm i.d) coated with 0.25um cyano propyl phenyl siloxane (ZB-1701) phase. The GC was temperature programmed from 50-310°C at 5°C min and held at final temperature for 10 minutes with helium as the carrier gas (flow rate of 1ml/min, initial pressure of 50kPa, split at 30 mls/min). The acquired data was later stored on DVD for later data processing, integration and printing. Peaks were identified and labelled after comparison of their mass spectra with those of the NIST05 library if fit was > 90% or from their elution order from the literature.

Cell culture

Rat B-13 hepatocyte progenitor cells were routinely expanded in low glucose (1000mg/l) Dulbecco's minimum essential medium (DMEM) supplemented with 10% (v/v) FCS, 80u/ml penicillin and 80µg/ml streptomycin. B-13 cells were converted into functional hepatocytes (B-13/H cells) in vitro through addition of 10nM dexamethasone essentially as previously outlined[52,53,23]. B-13/H cells are a non-proliferative functional hepatocyte-like cell expressing a variety hepatic functions (such as functional cytochrome P450s) at near normal liver levels[54]. The human H69 cholangiocyte cell line[55] was routinely expanded in 3:1 (v/v) ratio of DMEM and Nutrient F12 Ham's medium supplemented with 180µM adenine, 2nM triiodothyronine, 5.5µM epinephrine, 1µM hydrocortisone, 10% v/v FCS, 1x Insulin/transferrin/selenium (Gibco) and 1 x Pen/Strep (Lonza). The human hepatoma HepG2 cell lines was cultured as previously described[56]. The human breast cancer MCF-7 cell line was cultured as previously described[57]. All cells were incubated at 37^oC in an humidified incubator gassed with 5% CO_2 in air. Human cholangiocytes were isolated from resected human liver using an immune-bead approach as previously described and cultured in 1:1 [v/v] DMEM:Hams F12 medium supplemented with 10% (v/v) FBS, 2 mM glutamine, 100 U/ml penicillin, 100 µg/ml streptomycin, 10 ng/ml epidermal growth factor (EGF), 0.248 IU/ml Insulin, 2 µg/ml hydrocortisone, 10 ng/ml cholera toxin, 2nM tri-iodo-L-thyronine and 5 ng/ml hepatocyte growth factor (HGF)[58]. Human hepatocytes were isolated from a 42 year old male donor by collagenase perfusion essentially as previously described[59] and cultured on collagen-coated plates in Williams medium E supplemented with 10% (v/v) FCS, 80u/ml penicillin, 80µg/ml streptomycin, 10nM dexamethasone and 1ug/ml insulin. After an overnight culture period, the medium was aspirated, the cells were washed 3 times with sterile PBS prior to incubation with M8OI in a short-term simplified incubation medium (STIM buffer: 0.10M NaCl, 5.4mM KCl, 0.34mM Na_2HPO_4 $12H_2O$, 0.44mM KH2PO4, 20mM glucose, 1mM $CaCl_2$, 40mM $NaHCO_3$, 4mM glutamine, 100µM L-alanine, 100µM L-asparagine, 100µM L-aspartic acid, 100µM L-glutamic Acid, 100µM glycine, 100µM L-proline and 100µM L-serine, pH 7.4 when gassed with 5% CO_2 in air) to minimise interference with M8OI detection, typically 1.5mls/well of a 6 well plate. As a control, M8OI was incubated identically in a cell-free culture vessel. After 24 hours, the

STIM incubation was removed, centrifuged at 13,000 rpm for 1 min and 10 volumes of supernatant clear of any cellular material retained and added to 1 volume 1% phosphoric acid. Acid-precipitated material was removed by centrifugation (13,000 rpm, 1min) and the supernatant was retained at stored at 4°C prior to analysis. Human tissue was obtained with patient consent and with approval of the Newcastle & North Tyneside 2 Research Ethics Committee.

Cell toxicity and proliferation assays

Thiazolyl blue tetrazolium bromide (MTT) reduction was used as a high throughput screen for cell viability by replacing culture media with fresh media containing 0.5mg/ml MTT and returning cells to the incubator for between 2-4 hours. The medium was then replaced with an equal volume of isopropanol and after mixing, absorbance was determined at 570nm (with background absorbance at 690nm also determined and reading subtracted from reading at 570nm). Results are expressed as percentage absorbance relative to vehicle treated cells. Trypan blue staining was carried out essentially as previously outlined[56]. The incorporation of ^{3}H thymidine in cells was carried out essentially as previously outlined[60]. Caspase activities were determined using an ApoTox-GloTriplex assay kit (Promega, Southampton, UK) following the manufacturer's instructions. Luminescence was determined using a Tecan infinite 200 plate reader (Männedorf, Switzerland) employing a 1 second integration time and normalised to GF-AFC fluorescence. Genomic DNA was examined for the laddering associated with apoptosis as previously described[56].

Receptor - luciferase reporter gene assays

Human AhR and metallothionein (MT1) activation was examined in Hep2G cells and a proprietary screening assay purchased from SABiosciences (Manchester, UK). Human ERα activation was examined using MCF-7 and an in-house luciferase reporter gene assay as previously described[61,57]. All data were normalised by co-transfection with the renilla encoding construct RL-TK (Promega, Southampton, UK). AR-EcoScreenTM cells were used to study human androgen receptor transcriptional activity, as described

in the OECD guideline no. 458 (OECD, 2016). The cells were purchased from National Institute of Biomedical Innovation, Health and Nutrition JCRB Cell Bank (Osaka, Japan). DMEM-F12 medium without phenol red was supplemented with 5% FBS (Gibco, Thermo Fisher Scientific, USA), 1% L-glutamine (Lonza, Switzerland), 100 U/mL penicillin, 100 µg/mL streptomycin (Lonza, Switzerland), 200 µg/mL zeocin (Gibco, Thermo Fisher Scientific, USA), and 100 µg/mL hygromycin (InvivoGen, France) and used for cell culturing. A 0.25% trypsin and 0.02 unit EDTA solution with phenol red (Sigma-Aldrich, USA) was used for sub-culturing cells twice per week. Medium was changed every 2-3 days. PPARα activation was also determined in HepG2 cells. HepG2 cells were seeded in white clear-bottomed 96-well plates (Corning, NY, USA) at a concentration of 15 000 cells/well. After 48 hours, medium was exchanged and cells were transfected with 30 ng/well of RL-TK plasmid. In addition, the cells for the PPARα reporter gene assay were transfected with 45 ng/well of a Gal4-human-PPARα-ligand binding domain-plasmid and 45 ng/well of UAS-Gal-Luc-plasmid (both generously provided by Dr Jan Fleckner, Novo Nordic, Denmark). All transfections were performed by use of 0.3 µL/well of Lipofectamine® 2000 Reagent (Invitrogen, Thermo Fisher Scientific, USA). After 24 hours transfection, cells were treated with soil extracts and positive controls. The experiments were finalized 24 hours later, with medium removal, cell lysis with 20 µL of passive lysis buffer from the Dual Luciferase assay kit (Promega, Southampton, UK) for at least 15 minutes. Luciferase activity measurements were performed with the Dual-Luciferase® Reporter Assay System (Promega, Southampton, UK) according to manufacturer's protocol.

Gene expression analyses

Cells were treated with extracts or a variety of control chemicals with separate appropriate solvent vehicles as controls. Total RNA was the isolated using TRIzol (Life Technologies, Paisley, UK), DNAse-treated using RQ1 DNAse (Promega, Southampton, UK) and complementary DNA synthesised using MML-V reverse transcriptase (Promega) following the manufacturer's instructions. Quantitative real-time PCR was carried out using SYBR green (Sigma) on a 7500 Fast thermocycler (Applied biosciences, Paisley, UK).

The rCyp1a1 and 18S rRNA primer sequences used are those as previously described[53]. Cyp1a1 and AMPK protein expression was determined by Western blotting essentially as outlined[53], with normalisation to β-actin protein expression. Anti-Cyp1a1, anti-AMPK and anti-phospho-AMPK (phosphorylated at Thr^{172}), and anti-β-actin antibodies were purchased from Daiichi Chemical Co (Tokyo, Japan), Cell Signalling Technologies (Leiden, The Netherlands) and Sigma (Poole, UK) respectively.

Seahorse analyses

Mitochondrial activity was assessed using the Seahorse XF analyser XFe24 or XFe96 (Seahorse Bioscience, Copenhagen, Denmark) following the manufacturer's guidelines. Calibration plates were incubated overnight at 37°C with calibrant solution prior to use. Oxygen consumption rate (OCR) and extracellular acidification rate (ECAR) measurements were undertaken using the default 3 cycles of 2-3-2 (minute wash-measure-read settings respectively) and readings for the last of these 3 cycles were used for data analysis. Cell number and concentrations of oligomycin, FCCP, rotenone and antimycin A were individually optimised in B-13 and H69 cells. The concentrations used were 1µM or 2µM FCCP for B-13 and H69 cells respectively and 1µM oligomycin and 0.5µM rotenone/antimycin A for both cell types. Fifty thousand and forty thousand cells/well were used for experiments with B-13 and H69 cells. When using B-13s in a XFe, 15 thousand cells/well were used for experiments. Assays were performed in assay medium (Seahorse Bioscience) supplemented with 5.5mM glucose and 1mM sodium pyruvate, adjusted to pH7.4. In extract screening assays, cells were pre-treated with the different environmental samples for 1 hour in the assay medium at 37°C and ambient CO_2 prior to measurement. Oxygen consumption rate (OCR) and extracellular acidification rat (ECAR) values were normalised to protein content in each well at the end of the experiment, calculated using Bradford reagent (Sigma, Poole, UK). Mitochondrial parameters were calculated from OCR data as follows: Basal respiration, Basal OCR – antimycin A/rotenone; ATP production, basal OCR - oligomycin OCR; maximal respiration, FCCP OCR – antimycin A/rotenone OCR.

Glucose assay

Culture medium glucose concentrations were determined using a glucose oxidase/horse radish peroxidase colorimetric assay as previously described[62].

ATP assay

Intracellular ATP content was determined using a CellTiter glo 2.0 kit (Promega, Southampton, UK) following the manufacturer's guidelines. Luminescence was read using a Tecan infinite 200 plate reader using a 1s integration time.

16S rRNA PCR

Extract samples (1µl) were screened for the presence of bacteria by direct PCR for 16S rRNA gene essentially as described[53] using upstream and downstream primers with the sequences 5'-AGAGTTTGATCCTGGCTCAG and 5'-GGTTACCTTGTTACGACTT respectively[63] (100% complementarity with 102 separate 16S rRNA bacteria and archaea species as determined via BLAST (https://blast.ncbi.nlm.nih.gov/Blast.cgi). PCR products were amplified over 35 cycles using an annealing temperature of 55°C (1 mins) and an extension temperature of 72°C (2 mins). A colony of E. coli TOP10 bacteria (ThermoFisher, Paisley, UK) was diluted 1/1000 in PBS buffer and 1µl used in the PCR reaction as a positive control.

SDS-PAGE/silver staining

SDS-PAGE was performed essentially as previously outlined[53] except that samples were prepared by mixing extract samples 1:1 (v/v) with loading buffer (120mM Tris (pH 6.8), 20% glycerol (v/v), 3.9% (w/v) SDS, 0.74% (w/v) bromophenol blue and 20mM DTT) and loaded onto 4% polyacrylamide stacking gel / 14% polyacrylamide separating gel. Following SDS-PAGE, gels were silver stained using the Pierce silver stain kit (Paisley, UK) following the manufacturer's protocol.

Spectrophotometry

Dual beam spectrophotometry was carried out using a Cary 300 spectrophotometer (Agilent, Stockport, UK) using the relevant solvent as the blank. Fluorescence spectrophotometry was carried out using LS50-B spectrophotometer (Perkin Elmer). PBS extract samples were excited at 220nm and emission between 250-900nm measured. Anthracene and anthraquinone solutions were excited at 254nm and emission between 280-900nm measured.

Chromatography and mass spectrometry

Chromatographic separation of the soil extracts was performed by gradient elution with an ACE C_{18} capillary LC column – 100mm x 300µm x 3µm (HighChrom)– fitted with a 0.25µm column saver pre-column filter, with (A) 0.1 % formic acid in water and (B) 0.1% formic acid in acetonitrile as mobile phase, at a flow rate of 5µL/min. Gradient conditions were 5 % B, held for 1 min; then increased to 95 % B over 40 min, held until 45 min and returned to 5 % B at 45.1 min and held until 50.0 min. Total run time was 50.0 min. The column and auto-sampler temperatures were 25°C and 8°C respectively.

Soil extracts were analysed using non-targeted data independent LC-MS/MS techniques. The particular data independent analysis method employed was Sequential Window Acquisition of all THeoretical fragment-ion spectra (SWATH) mass spectrometry (MS), which utilises the very fast scanning speeds of quadrupole time-of-flight (QTOF) mass spectrometers. SWATH MS (Sciex, Framingham, MA) is a form of data-independent analysis that repeatedly cycles through consecutive pre-set precursor ion isolation windows, detecting all fragment ion spectra from all the precursor ions contained in a specific window at a given time, providing highly selective MS/MS mass spectra of all analytes. Protonated molecular ions were detected via a time-of-flight (TOF) MS scan covering the 100–800 Da mass range. The TOF MS scan was followed by SWATH MS/MS acquisition, acquired in high sensitivity mode at a mass resolution of at least 20,000, with a collision energy spread of 30 ± 15V over a mass range of 30–825 Da, using 20 Da SWATH

isolation windows. Mass calibration was performed on every second sample by injection of a calibration solution through the LC-MS/MS system.

LC-MS/MS data were processed using MasterView software version 2.2 with SWATH MicroApp version 2.0 (Sciex, Framingham, MA). Analyte identification was performed on TOF MS data with LibraryView version 1.0 (Sciex, Framingham, MA) and ChemSpider Library version 2.0 (Royal Society of Chemistry, Cambridge, UK), integrated within the MasterView software. Empirical formulae with a mass error of less than 0.5ppm were considered as viable candidates, which were interrogated against the ChemSpider library. The TOF MS and MS-MS spectra was matched against postulated structures using theoretical molecular mass fragmentation calculation algorithm within the MasterView software.

M8OI and metabolites were identified using non targeted data independent LC-HR-MS/MS techniques using a TripleTOF 5600 high-resolution quadrupole time-of-flight (TOF) mass spectrometer (Sciex) equipped with a DuoSpray ion source operated in positive electrospray mode, coupled to an Eksigent Nano LC 420 system. AnalystTF version 1.7.1 was used for instrument control and data acquisition. Chromatographic separation was achieved by gradient elution with an ACE C_{18} capillary liquid chromatography column (100mm x 300µm x 3µm; HighChrom) fitted with a 0.25 µm column saver pre-column filter, using (A) 0.1% formic acid in water and (B) 0.1% formic acid in acetonitrile as mobile phase, at a flow rate of 5µL/min. Gradient conditions were 5% B, held for 1 min, then increased to 95% B over 40 min, held at 95% B until 45 min, returned to 5% B at 45.1 min, and held until 50.0 min. The column and autosampler temperatures were 30 °C and 12 °C, respectively. Data processing for the identification of M8OI and metabolites was performed using MasterView software version 1.1 incorporated within PeakView Software version 2.2(Sciex). M8OI and metabolites were quantified by standard multiple reaction monitoring (MRM) techniques using a Q-Trap 5500 hybrid linear ion trap/triple quadrupole mass spectrometer (Sciex) coupled to a Shimadzu Prominence liquid chromatograph. Analyst version 1.6.2 and MultiQuant version 2.0 (Sciex) were used for instrument control/data acquisition and quantitative analysis respectively. Chromatographic separation was achieved by gradient elution with a Raptor Biphenyl

chromatography column (100 mm x 2.1 mm x 2.7 µm; Restek) equipped with a guard column containing identical packing material, with (A) 0.1% formic acid in water and (B) 0.1% formic acid in methanol as mobile phase, at a flow rate of 400 µL/min. Gradient conditions were 5% B, for 1 min, then we increased to 95% B over 25 min, held until 30 min and returned to 5% B at 30.1 min, and held until 35.0 min. The column and autosampler temperatures were 50 °C and 12 °C, respectively with 2µL injection volume per sample. MRM transitions for M8OI, HOM8OI and COOHM8OI were 195.2 -> 83.1, 211.2 -> 83.1 and 225.2 -> 83.1 respectively, with a collision energy of 30V.

NMR spectroscopy

^{1}H, ^{13}C, and various 2D (including ^{15}N HMBC) NMR spectra were obtained at 700.13 (^{1}H) 176.07 (^{13}C) and 70.95 (^{15}N) MHz on a Bruker Avance III HD 700 MHz NMR spectrometer equipped with a Prodigy TCI cryoprobe. A ^{14}N NMR spectrum was obtained on a Bruker Avance III HD 500 MHz NMR spectrometer at 36.14 MHz. ^{1}H and ^{13}C chemical shifts were referenced to TMS, ^{14}N and ^{15}N chemical shifts to nitromethane. NMR spectra were obtained in both CD_3OD and $CDCl_3$. After dissolution in CD_3OD, signals attributable to formic acid or the formate anion were evident in the proton and ^{13}C NMR spectra. Some of the material was insoluble in $CDCl_3$. It was found that several of the chemical shifts were sensitive to the solvent used and the nature of any counter ion. This affected comparison with literature data, and the spectral assignments were therefore confirmed from COSY, HSQC and HMBC (both ^{13}C and ^{15}N) experiments. In particular, it was found that the proton H^2 showed significant acidity and was exchanged for deuterium in CD_3OD masking the C^2 resonance in this solvent.

Animal study

Adult female mice C57Bl6 mice bearing a transgene composed of three NF-κB sites from the Ig κlight chain promoter coupled to the gene encoding firefly luciferase[64] (age 15-16 months; 5 control animals, 6 M8OI-treated animals) were administered M8OI in their drinking water such that they received 1mg/kg body weight per day calculated using the EFSA default value for drinking water consumption[65]. At 14 and 28 days, exposure to M8OI was sequentially increased to 2 and 4 mg/ kg body weight per day respectively. Mice were killed by cervical dislocation at 45 days and blood collected for clinical chemistry analyses. Bile was by insertion of a needle attached to a syringe into the gall bladder followed by gentle aspiration. No adverse effects to M8OI were observed based on comparison of body weights, luciferase expression, liver pathology (H&E stained sections) and serum liver enzyme (ALT, ALP) levels compared to controls. This study was performed under a UK Home Office licence with Local Ethics Committee approval.

Assay to determine the incorporation of lipoic acid or xenobiotics into the E2 component of PDC

Incorporation of lipoic acid or xenobiotics into the E2 component of pyruvate dehydrogenase via the exogenous (scavenging) lipoylation pathway was determined essentially as previously determined[27]. In brief, recombinant bovine lipoate activating enzyme (LEA) and lipoyl-AMP(GMP):N-lysine lipoyl transferase (LT) were expressed and unlipoylated PDC-E2-ILD (Ulip) purified by anion exchange chromatography as previously described. For a typical assay, 10µg of the LAE/LT preparation and 10µg ULip were mixed in a 50µl reaction mixture containing 20mM Tris-HCl (pH 7.5), 40mM potassium phosphate (pH 7.8), 4mM MgCl2, BSA (0.3mg/ml) and 4mM GTP. Lipoylation was initiated through the addition of lipoic acid (DL-6,8-thioctic acid (Sigma-Aldrich Company, Dorset, UK) or up to 20mM xenobiotic. COOH7IM was custom synthesized by Fountainbridge, Edinburgh, UK (see **Supplementary Fig. 11** for analytical data). Reactions were incubated at 37°C for up to 300 minutes to determine the point of full lipoylation. Reactions were terminated by boiling for 1min followed by processing for immunoblotting after suspending samples in non-denaturing sample buffer and subjecting the samples to

PAGE on 15% non-denaturing gels. Separated proteins were transferred electrophoretically to Immobilon-P nitrocellulose membrane (Millipore, Herts, UK). Membranes were blocked with 5% (w/v) skimmed milk powder in PBS for 1 hour and the membranes washed in PBS + 0.05% (v/v) Tween 20. Membranes were probed with serum from patients with high AMA (anti-PDC) and incubated for 2 hours at room temperature followed by a 1 hour incubation using an anti-human IgG peroxidase-conjugated secondary antibody. After extensive washing, peroxidase reactivity was detected by enhanced chemiluminescence (Perbio Science Ltd, UK).

Statistical Analysis

For the comparison between two groups, an unpaired Students t-test was carried out and significance assumed where $p<0.05$. For comparison of multiple groups, ANOVA was carried out and where significant, differences between groups were determined using Bonferroni-Holm method. Where $p<0.05$, a significant difference was assumed.

SUPPLEMENTARY FIGURES

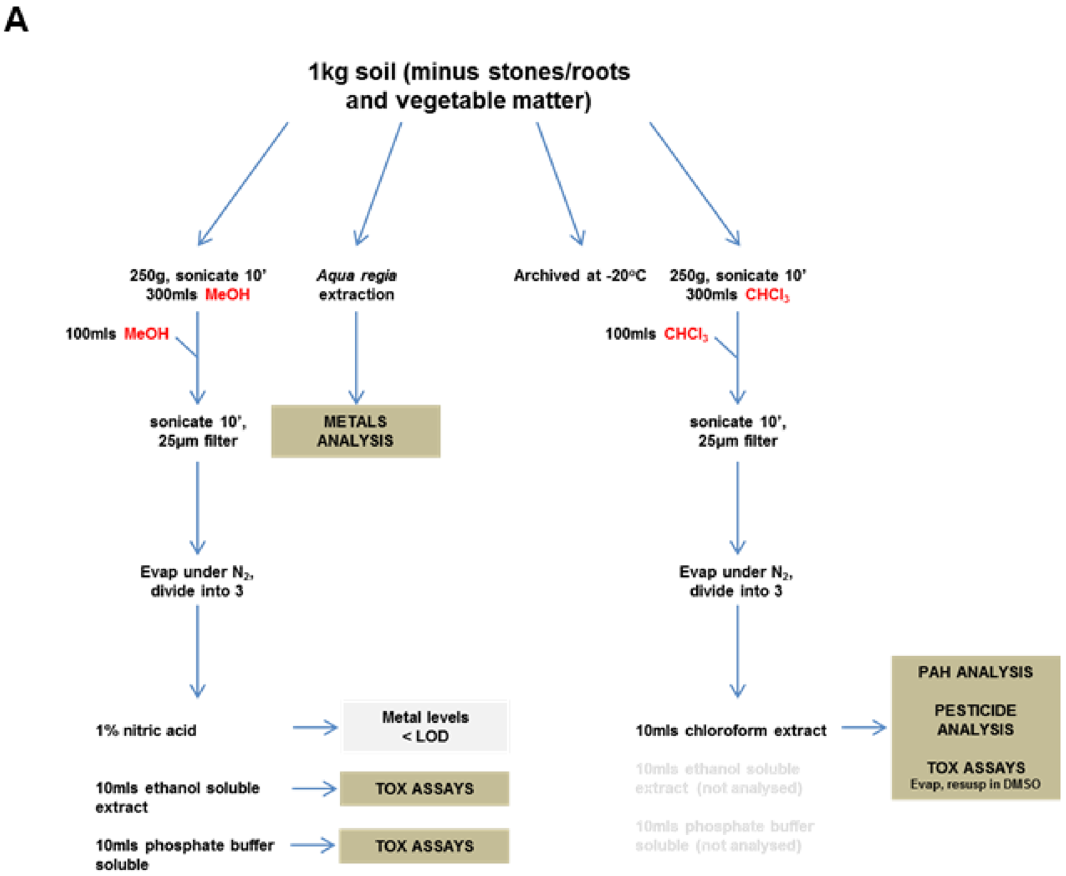

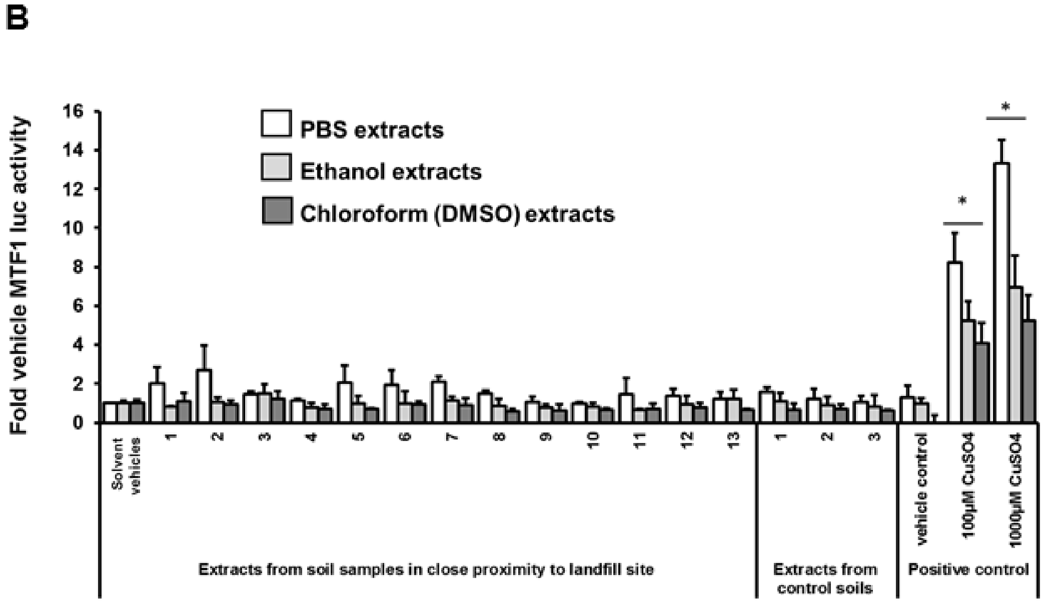

Fig. S1. Soil sample methodology and screening for heavy metals using a reporter gene approach. A, schematic diagram outlining the approach taken with regard to soil samples at each landfill or control sampling site. Metal concentrations were determined following aqua regia extraction and data are provided in Supplementary Table 1. Polyaromatic hydrocarbon (PAH) analysis data are provided in Supplementary Table 2. Note, chloroform solvent was not appropriate for use in cell based toxicity assays due to solvation of cultureware and therefore after its evaporation, the solid material was dissolved in the same volume of DMSO. **B**, Metallothionein TRE-luciferase reporter gene assay for the detection of heavy metals in all extracts. HepG2 cells were transfected with Cignal MTF1 Reporter (SABiosciences) and 24 hours later, exposed to 0.1% (v/v) of each extract. After a further 24 hours, cells were harvested and luciferase activities (normalised to constitutively expressed renilla activity present on the luciferase-encoding construct) were determined. Data are the mean and SD of 3 separate treatments for the same experiment typical of at least 3 separate experiments. *Significantly different from vehicle control ($p < 0.05$) using ANOVA/Bonferroni-Holm comparison between groups.

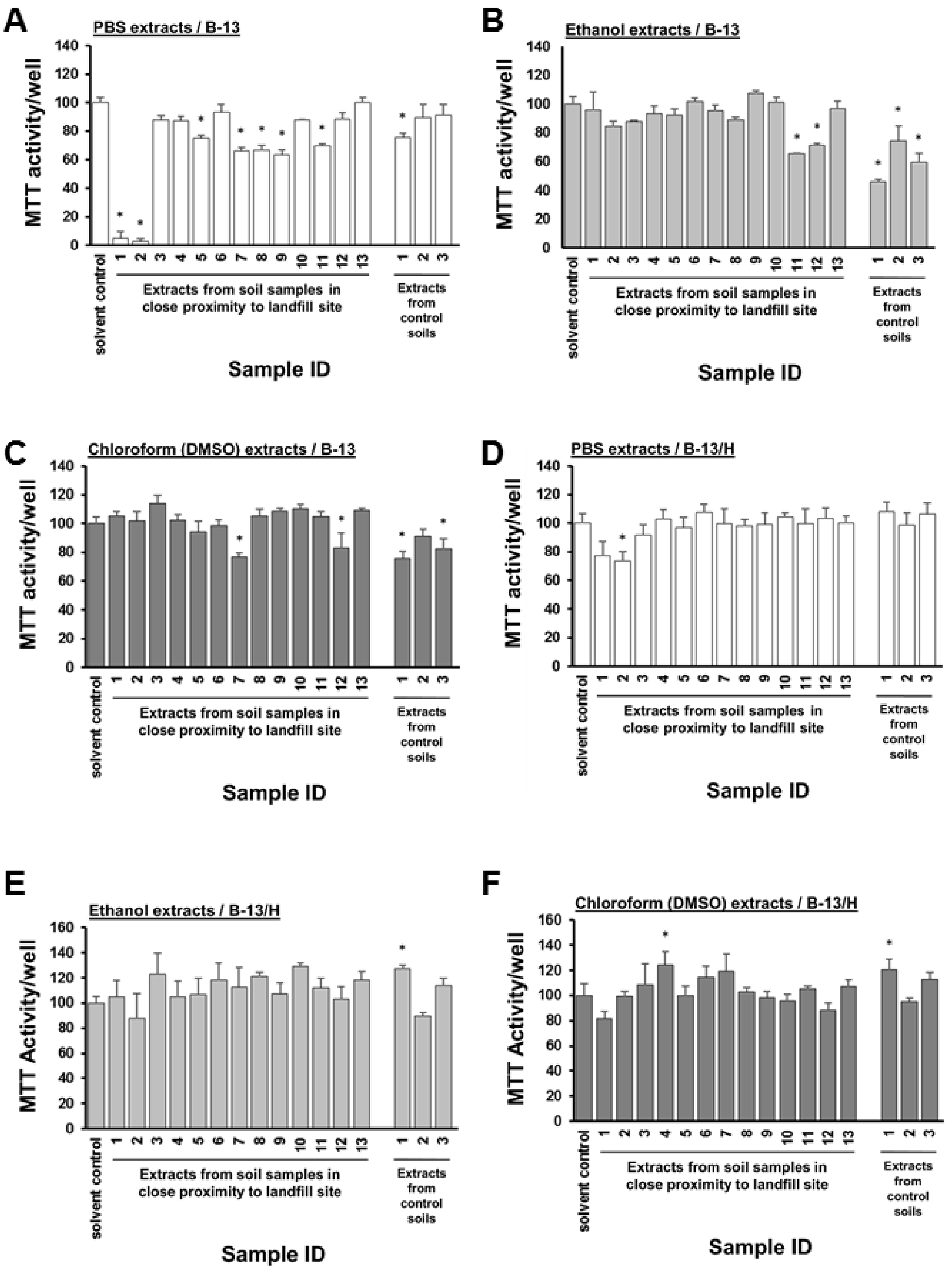
A
PBS extracts / B-13
B
Ethanol extracts / B-13
C
Chloroform (DMSO) extracts / B-13
D
PBS extracts / B-13/H
E
Ethanol extracts / B-13/H
F
Chloroform (DMSO) extracts / B-13/H
MTT activity/well
MTT Activity/well
solvent control
Extracts from soil samples in close proximity to landfill site
Extracts from control soils
Sample ID

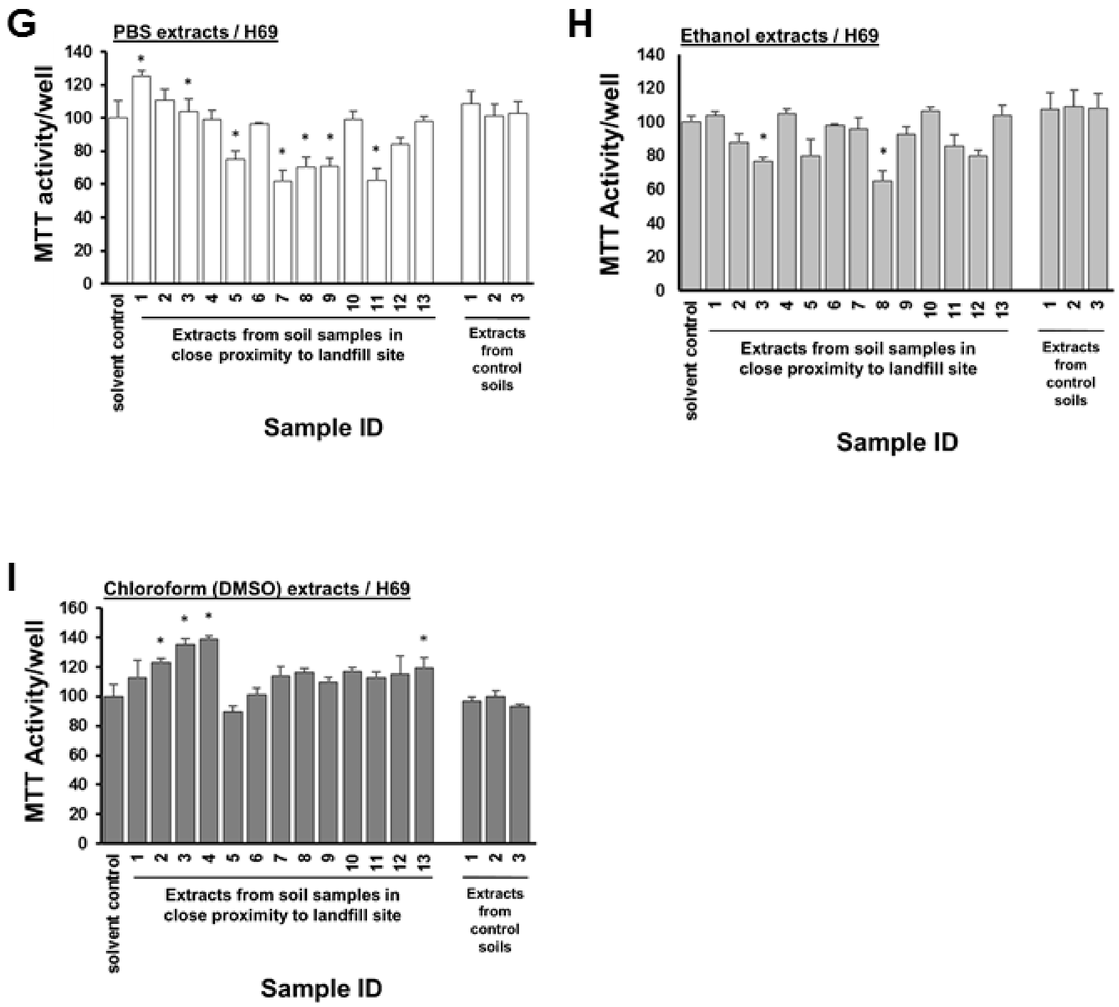

Fig. S2. Toxicity of soil samples in the hepatic progenitor B-13, hepatocyte-like B-13/H and human H69 cholangiocyte cells. MTT reduction in the indicated cell type with the indicated soil extracts (at 1% (v/v)) following 24 hour exposure. Data are the mean and SD of 3 separate treatments for the same experiment typical of at least 3 separate experiments and are expressed as a percentage of 1% (v/v) solvent control vehicle. *Significantly different from solvent control ($p < 0.05$) using ANOVA/Bonferroni-Holm comparison between groups.

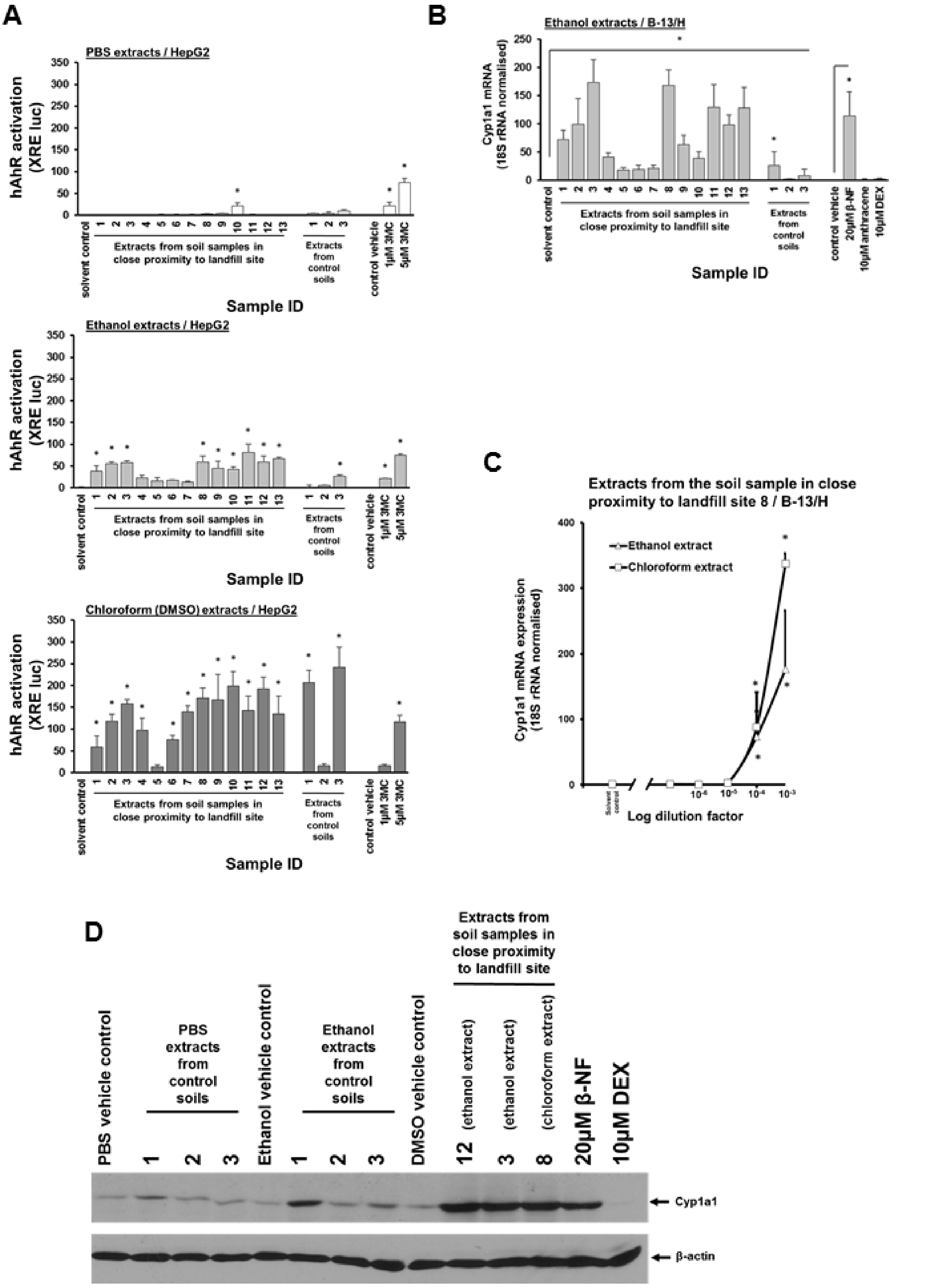
A
PBS extracts / HepG2
hAhR activation (XRE luc)
Extracts from soil samples in close proximity to landfill site
Extracts from control soils
solvent control
control vehicle
1µM 3MC
5µM 3MC
Sample ID
Ethanol extracts / HepG2
Chloroform (DMSO) extracts / HepG2
B
Ethanol extracts / B-13/H
Cyp1a1 mRNA (18S rRNA normalised)
20µM β-NF
10µM anthracene
10µM DEX
C
Extracts from the soil sample in close proximity to landfill site 8 / B-13/H
Ethanol extract
Chloroform extract
Cyp1a1 mRNA expression (18S rRNA normalised)
Log dilution factor
D
Extracts from soil samples in close proximity to landfill site
PBS vehicle control
PBS extracts from control soils
Ethanol vehicle control
Ethanol extracts from control soils
DMSO vehicle control
12 (ethanol extract)
3 (ethanol extract)
8 (chloroform extract)
20µM β-NF
10µM DEX
Cyp1a1
β-actin

Fig. S3. Soil samples in the north east of the UK contain biologically active levels of AhR activating chemicals. A, human AhR activation (XRE-luc reporter gene) activities in HepG2 cells treated with 0.1% (v/v) of the indicated landfill or control site soil PBS (upper), ethanol (middle) or chloroform extracts, expressed as fold solvent vehicle controls. Data are the mean and SD of 3 separate determinations. 3MC, 3 methylcholanthrene, a transcriptional activator of the AhR[59]. **B**, qRT-PCR for Cyp1a1 mRNA transcript levels in B-13/H hepatocyte-like cells treated with 0.1% (v/v) of the indicated landfill waste site or control site soil ethanol extract, expressed as fold ethanol vehicle control. Data are the mean and SD of 3 separate determinations. β-NF, β-naphthoflavone, a transcriptional activator of the AhR[59], DEX, dexamethasone which at this concentration activates both the rat GR and PXR[66] and demonstrates selectivity of the assay for AhR activation rather than differences in B-13/H differentiation. **C**, dose-response effect for Cyp1a1 mRNA transcript levels in B-13/H hepatocyte-like cells as determined via qRT-PCR after treatment with the indicated dilution of landfill waste site soil ethanol extract, expressed as fold ethanol vehicle control. Data are the mean and SD of 3 separate determinations. **D**, Western blot for Cyp1a1 expression in B-13/H cells treated with 0.1% (v/v) of the indicated landfill waste site or control site soil extract daily (for 3 days) and harvested for analysis on day 4. [*]Significantly different from solvent control (for soil extracts) or control vehicle for known chemicals ($p < 0.05$) using ANOVA/Bonferroni-Holm comparison between groups.

A

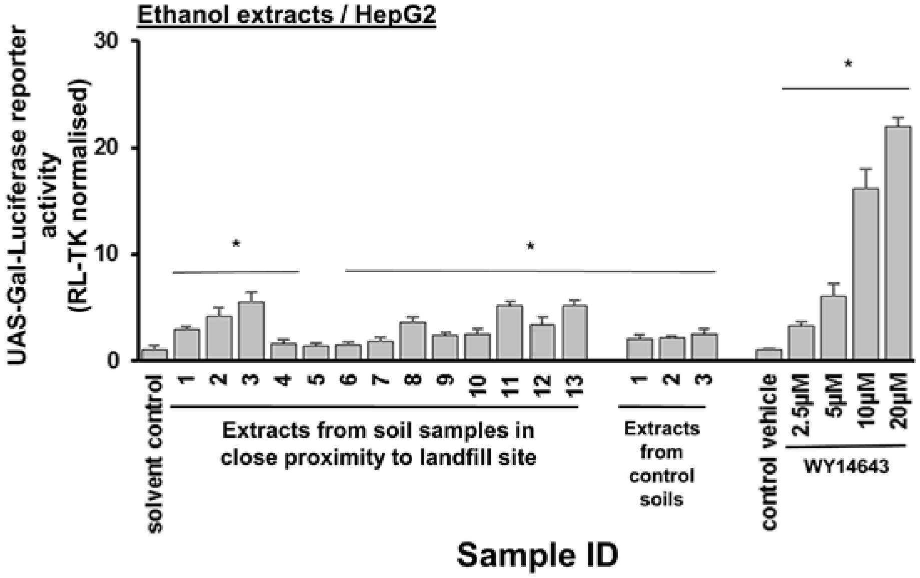

B

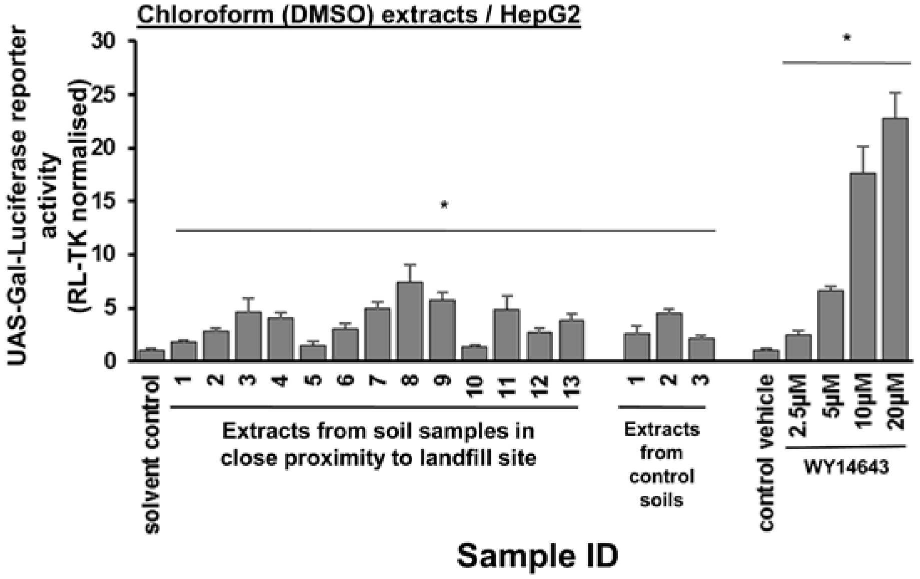

Fig. S4. Soil samples in the north east of the UK contain biologically active levels of peroxisome proliferator activated receptor-α (PPARα) activating chemicals. Human PPARα activation (UAS-Gal-Luciferase). HepG2 cells were transfected with reporter constructs and 24 hours later, treated with either 0.1% (v/v) of ethanol soil extracts (**A**) or 0.1% (v/v) chloroform extracts after reconstitution in DMSO (**B**).

After 24 hours exposure, reporter gene activities were determined as outline in the methods section with data the mean and SD of at least 3 separate transfections. WY14643 is a PPARα activator[67]. *Significantly different normalised luciferase activity from solvent control or control vehicle ($p < 0.05$) using ANOVA/Bonferroni-Holm comparison between groups.

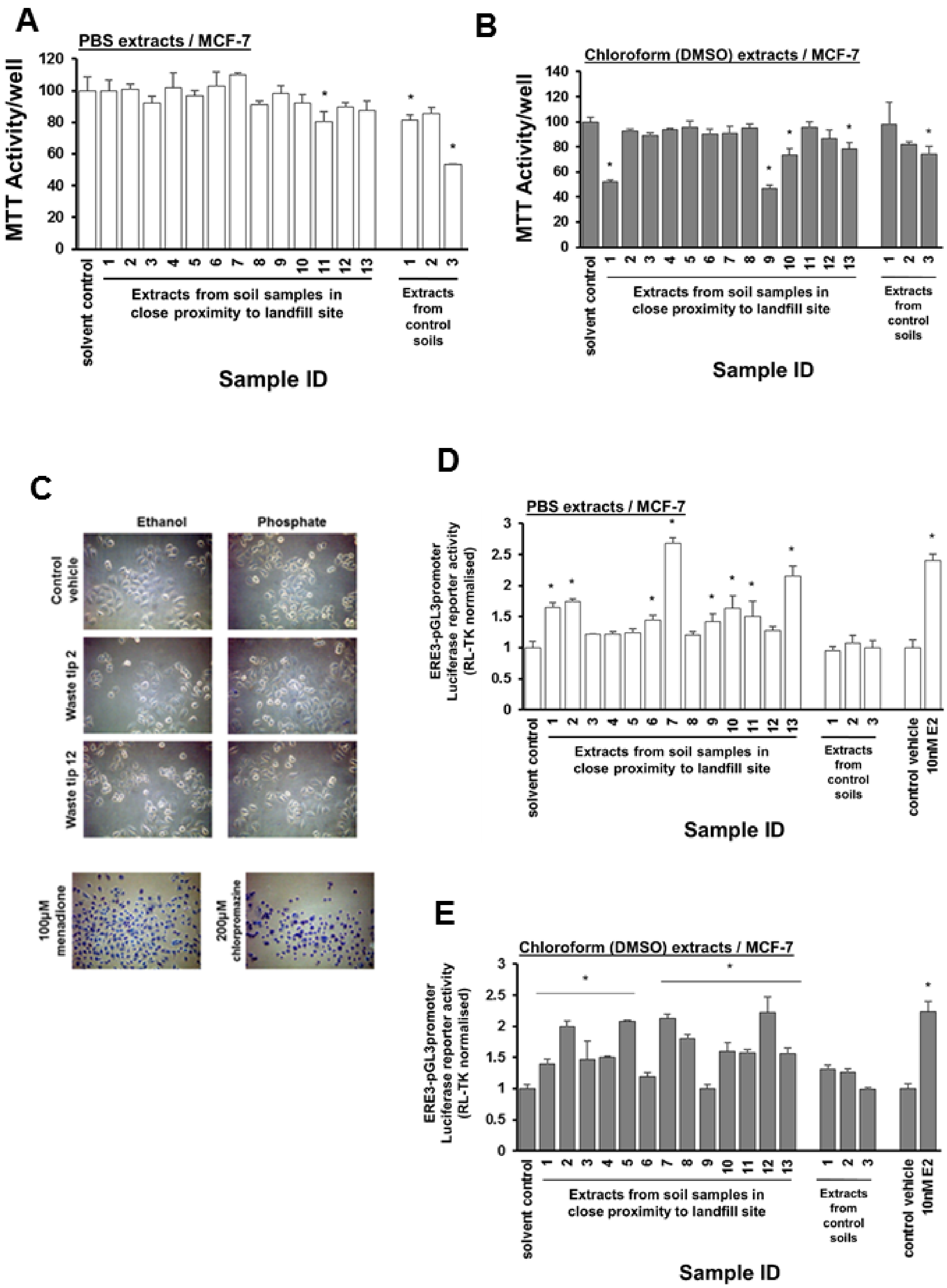
A
PBS extracts / MCF-7
MTT Activity/well
solvent control
Extracts from soil samples in close proximity to landfill site
Extracts from control soils
Sample ID
B
Chloroform (DMSO) extracts / MCF-7
MTT Activity/well
solvent control
Extracts from soil samples in close proximity to landfill site
Extracts from control soils
Sample ID
C
Ethanol
Phosphate
Control vehicle
Waste tip 2
Waste tip 12
100μM menadione
200μM chlorpromazine
D
PBS extracts / MCF-7
ERE3-pGL3promoter Luciferase reporter activity (RL-TK normalised)
solvent control
Extracts from soil samples in close proximity to landfill site
Extracts from control soils
control vehicle
10nM E2
Sample ID
E
Chloroform (DMSO) extracts / MCF-7
ERE3-pGL3promoter Luciferase reporter activity (RL-TK normalised)
solvent control
Extracts from soil samples in close proximity to landfill site
Extracts from control soils
control vehicle
10nM E2
Sample ID

Fig. S5. Toxicity and estrogenicity of soil extracts in human MCF-7 cells. A, MTT reduction in MCF-7 cells following 24 hour exposure with the indicated PBS soil extracts (at 0.1% (v/v)). Data are the mean and SD of 3 separate determinations and are expressed as a percentage of 0.1% (v/v) solvent control vehicle. **B,** MTT reduction in MCF-7 cells following 24 hour exposure with the indicated chloroform soil extracts (at 0.1% (v/v)). Data are the mean and SD of 3 separate determinations and are expressed as a percentage of 0.1% (v/v) solvent control vehicle. *Significantly different MTT activity from solvent control ($p < 0.05$) using ANOVA/Bonferroni-Holm comparison between groups.

C, Trypan blue exclusion in MCF-7 cells after treatment with the indicated soil extract or positive (toxic) controls menadione or chlorpromazine. **D,** human ERα activation (ERE_3-pGL3promoter-Luc). MCF-7 cells were transfected with reporter constructs and 24 hours later, treated with 0.1% v/v of the PBS extracts. After 24 hours exposure, reporter gene activities were determined as outline in the methods section with data the mean and SD of 3 separate transfections. **E,** human ERα activation (ERE_3-pGL3promoter-Luc). MCF-7 cells were transfected with reporter constructs and 24 hours later, treated with 0.1% v/v of the chloroform extracts. After 24 hours exposure, reporter gene activities were determined as outline in the methods section with data the mean and SD of 3 separate transfections. *Significantly different normalised luciferase activity from solvent control ($p < 0.05$) using ANOVA/Bonferroni-Holm comparison between groups.

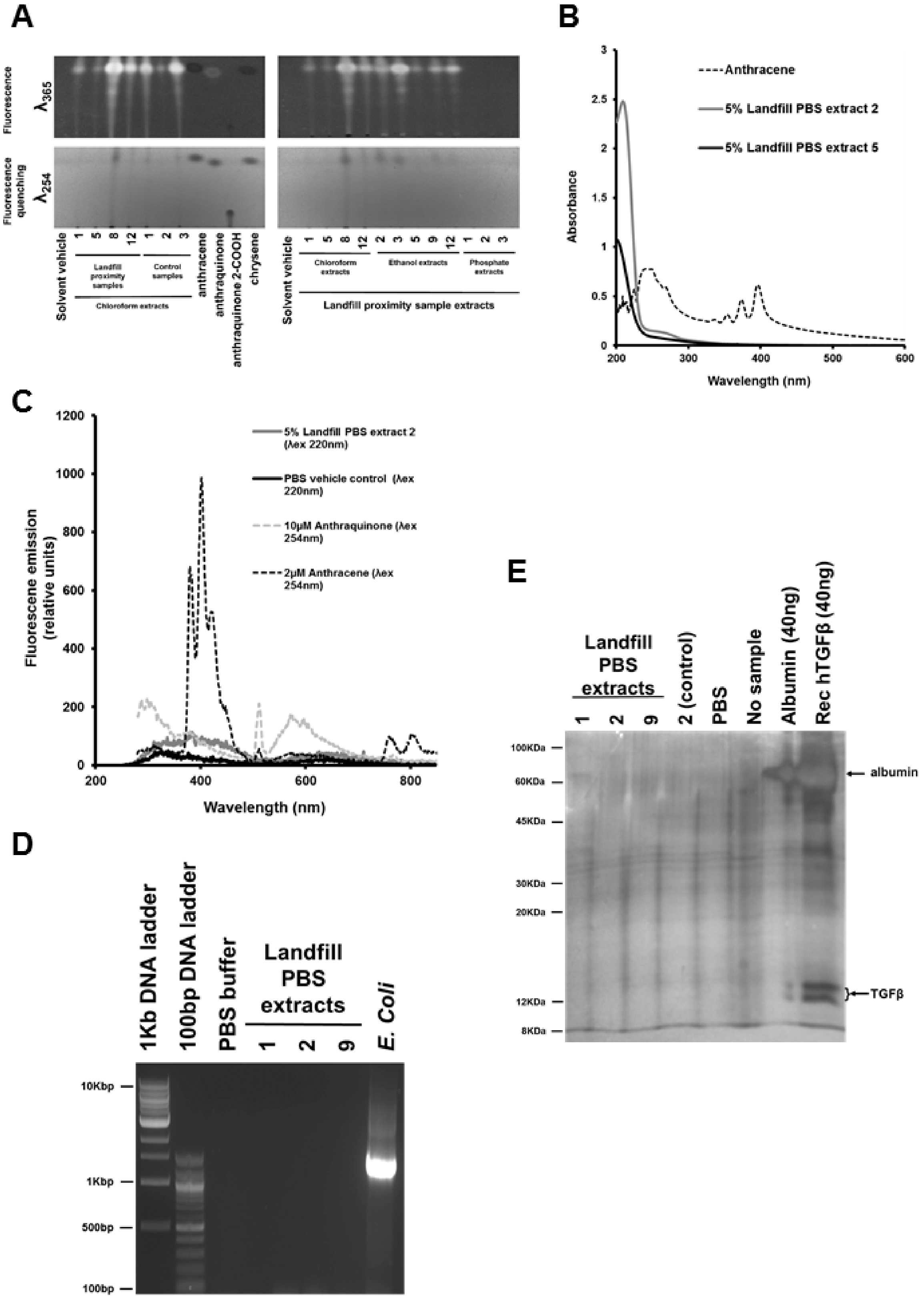
A
Fluorescence λ365
Fluorescence quenching λ254
Solvent vehicle
Landfill proximity samples
Control samples
Chloroform extracts
anthracene
anthraquinone
anthraquinone 2-COOH
chrysene
Chloroform extracts
Ethanol extracts
Phosphate extracts
Landfill proximity sample extracts
B
Anthracene
5% Landfill PBS extract 2
5% Landfill PBS extract 5
Absorbance
Wavelength (nm)
C
5% Landfill PBS extract 2 (λex 220nm)
PBS vehicle control (λex 220nm)
10μM Anthraquinone (λex 254nm)
2μM Anthracene (λex 254nm)
Fluorescene emission (relative units)
Wavelength (nm)
D
1Kb DNA ladder
100bp DNA ladder
PBS buffer
Landfill PBS extracts
E. Coli
10Kbp
1Kbp
500bp
100bp
E
Landfill PBS extracts
2 (control)
PBS
No sample
Albumin (40ng)
Rec hTGFβ (40ng)
100KDa
60KDa
45KDa
30KDa
20KDa
12KDa
8KDa
albumin
TGFβ

Fig. S6. Analysis of landfill PBS extract samples 1 and 2 containing a chemical(s) toxic to B-13 hepatic progenitor cells. A, TLC analysis of 10µl of the indicate samples or pure chemicals. Upper panels identify fluorescent chemicals, lower panels, chemicals capable of quenching silica fluorophore fluorescence. **B,** absorbance spectra for the indicated extracts, landfill site 2 PBS extract had an absorbance maximum at a wavelength of 221nm. **C,** Fluorescence emission spectra of the indicated extracts excited at the wavelengths as indicated. **D,** PCR analysis for the presence of bacterial contamination in landfill PBS extracts using primers designed to amplify a portion of the bacterial 16S rRNA. **E,** SDS-PAGE of 10µl of landfill PBS extracts followed by silver staining to screen for the presence of potential protein contamination in extracts. Note recombinant human TGFβ (rec hTGFβ) was supplied with excess albumin as a carrier protein.

Fig. S7. Nuclear magnetic resonance (NMR) spectroscopic analyses of pooled landfill PBS extracts 1 and 2 after HPLC purification of the peak associated with toxicity in B-13 cells.

Identification via NMR of $C_{12}H_{22}N_2$ chemical species in soil PBS extracts as [3-methyl-1-octyl-1H-imidazol-3-ium]$^+$ The material supplied was *ca*. 1 mg of a grey solid which had been previously subjected to various processes. Residual solvents which may have included formic acid were removed by freeze drying. ^1H, ^{13}C, and various 2D (including ^{15}N HMBC) NMR spectra were obtained at 700.13 (^1H) 176.07 (^{13}C) and 70.96 (^{15}N) MHz on a Bruker Avance III HD 700 MHz NMR spectrometer equipped with a Prodigy TCI cryoprobe. A ^{14}N NMR spectrum was obtained on a Bruker Avance III HD 500 MHz NMR spectrometer at 36.14 MHz. ^1H and ^{13}C chemical shifts were referenced to TMS and ^{14}N and ^{15}N chemical shifts to nitromethane. NMR spectra were obtained in both CD_3OD and $CDCl_3$. After dissolution in CD_3OD, signals attributable to formic acid or the formate anion were evident in the proton and ^{13}C NMR spectra. Some of the material was insoluble in $CDCl_3$. It was found that several of the chemical shifts were sensitive to the solvent used and the nature of any counterion. This affected comparison with literature data, and the spectral assignments were therefore confirmed from COSY, HSQC and HMBC (both ^{13}C and ^{15}N) experiments. In particular, it was found that the proton H^2 showed significant acidity and was exchanged for deuterium in CD_3OD; this made it difficult to observe the C^2 resonance in this solvent.

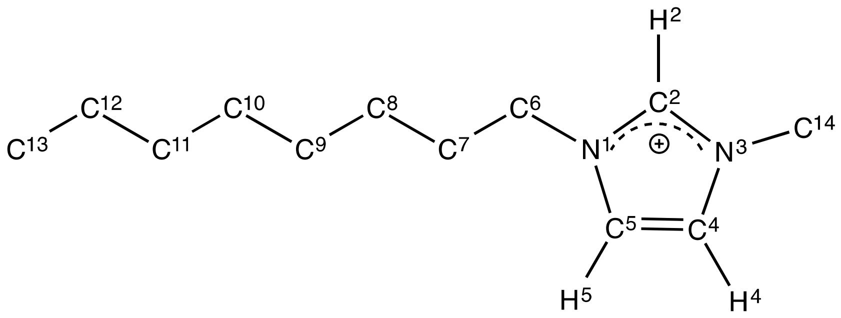

Key features of the assignment are:

1) A ^{15}N HMBC correlation of H^7 of the octyl chain with N^1 but not with N^3.

2) ^{15}N HMBC correlations of comparable intensity of H^2 with N^1 and N^3.
3) The relatively small nitrogen chemical shift difference of 11.8 ppm between N^1 and N^3 from the ^{15}N- HMBC and the direct ^{14}N spectra, which supports an imidazolium cation rather than an uncharged species.
4) The ready exchange of H^2 in methanol referred to above which again supports a cationic species.
5) Various HMBC correlations of H^2 with C^4, C^5, C^6 and C^{14}.
6) HMBC correlations of H^{14} with C^2 and C^4, but not C^5.
7) HMBC correlations of H^6 with C^2 and C^5, but not C^4.

The only significant differences between our assignments and those in the literature relate to the ^{13}C chemical shifts of C^7 and C^8 which we believe should be reversed, and to the chemical shifts of the ring protons which are very susceptible to the nature of the counter ion. These are minor discrepancies and we are confident of our assignments.

^1H NMR (700.13 MHz, $CDCl_3$) δ ppm: 0.87 (t, $^3J_{HH}$ = 7.2 Hz, 3H, H13), 1.20-1.39 (unresolved multiplets, 10H, H8-12), 1.91(quin, $^3J_{HH}$ = 7.4 Hz, 2H, H7), 4.12 (s, 3H, H14), 4.30 (t, $^3J_{HH}$ = 7.4 Hz, 2H, H6), 7.16 (m, 1H, H5), 7.18 (m, 1H, H4), 11.14 (s, 1H, H2).

$^{13}C\{^1H\}$ NMR (176.07 MHz, $CDCl_3$) δ ppm: 14.2 (C13), 22.7 (C12), 26.4 (C11), 29.06 (C10), 29.14 (C9), 30.5 (C7), 31.8 (C8), 36.8 (C14), 50.4 (C6), 121.3 (C5), 122.7 (C4), 139.6 (C2).

^{14}N NMR (36.14 MHz, $CDCl_3$) δ ppm: -208.9 (N3), -197.1 (N1).

^{15}N NMR (70.95 MHz, CD_3OD) δ ppm: -209.0 (N3), -196.2 (N1).

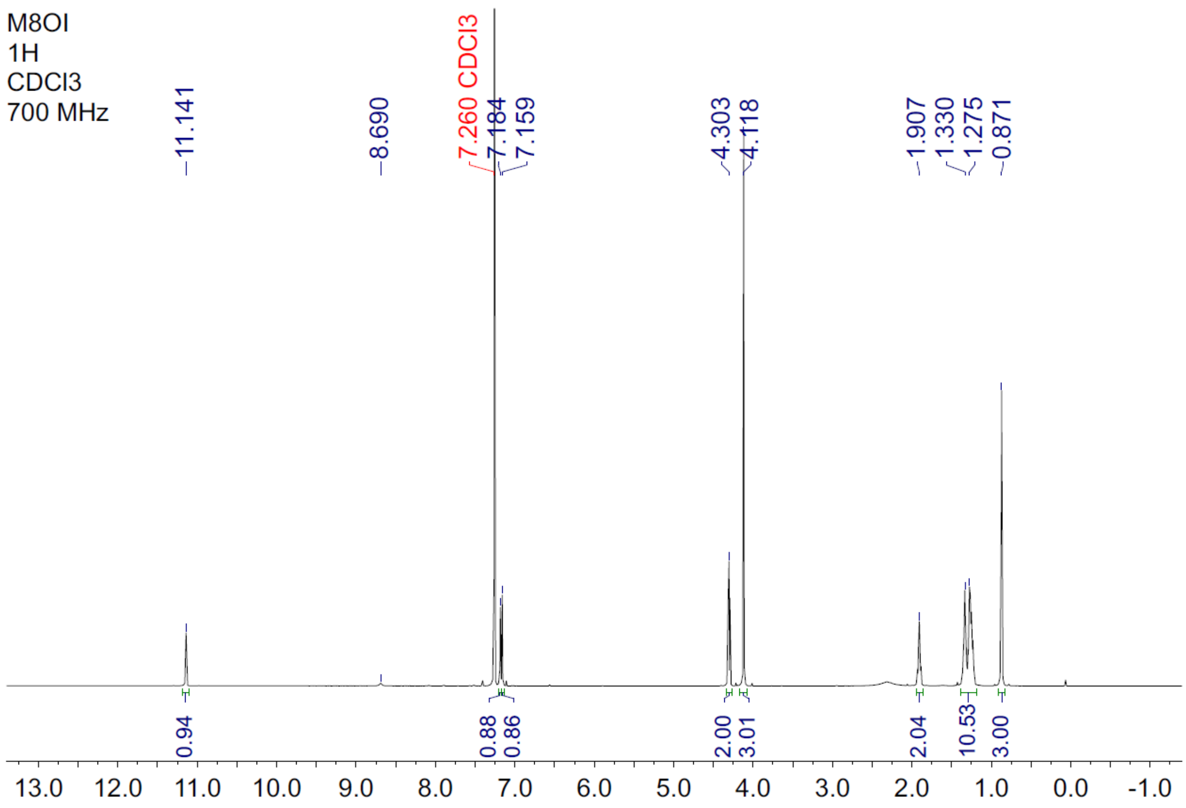
M8OI
1H
CDCl3
700 MHz
11.141
8.690
7.260 CDCl3
7.184
7.159
4.303
4.118
1.907
1.330
1.275
0.871
0.94
0.88
0.86
2.00
3.01
2.04
10.53
3.00
13.0 12.0 11.0 10.0 9.0 8.0 7.0 6.0 5.0 4.0 3.0 2.0 1.0 0.0 -1.0

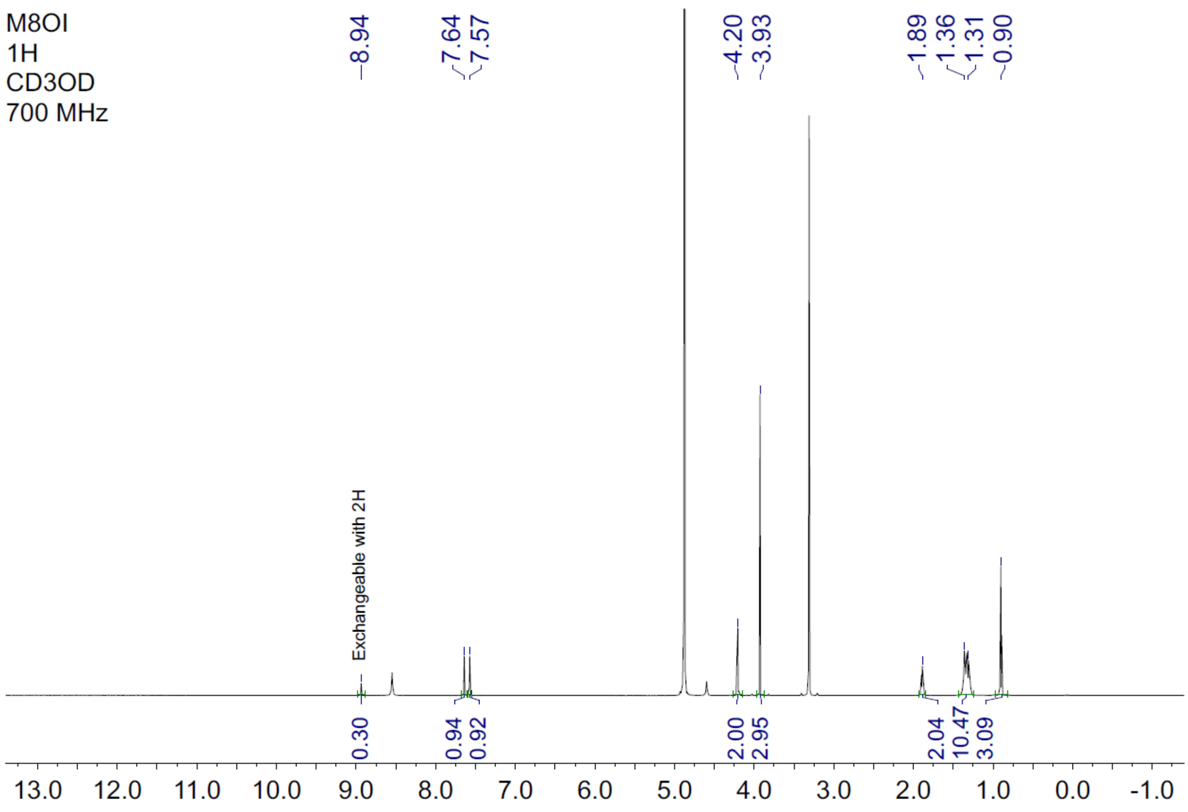
M8OI
1H
CD3OD
700 MHz
8.94
7.64
7.57
4.20
3.93
1.89
1.36
1.31
0.90
Exchangeable with 2H
0.30
0.94
0.92
2.00
2.95
2.04
10.47
3.09
13.0 12.0 11.0 10.0 9.0 8.0 7.0 6.0 5.0 4.0 3.0 2.0 1.0 0.0 -1.0

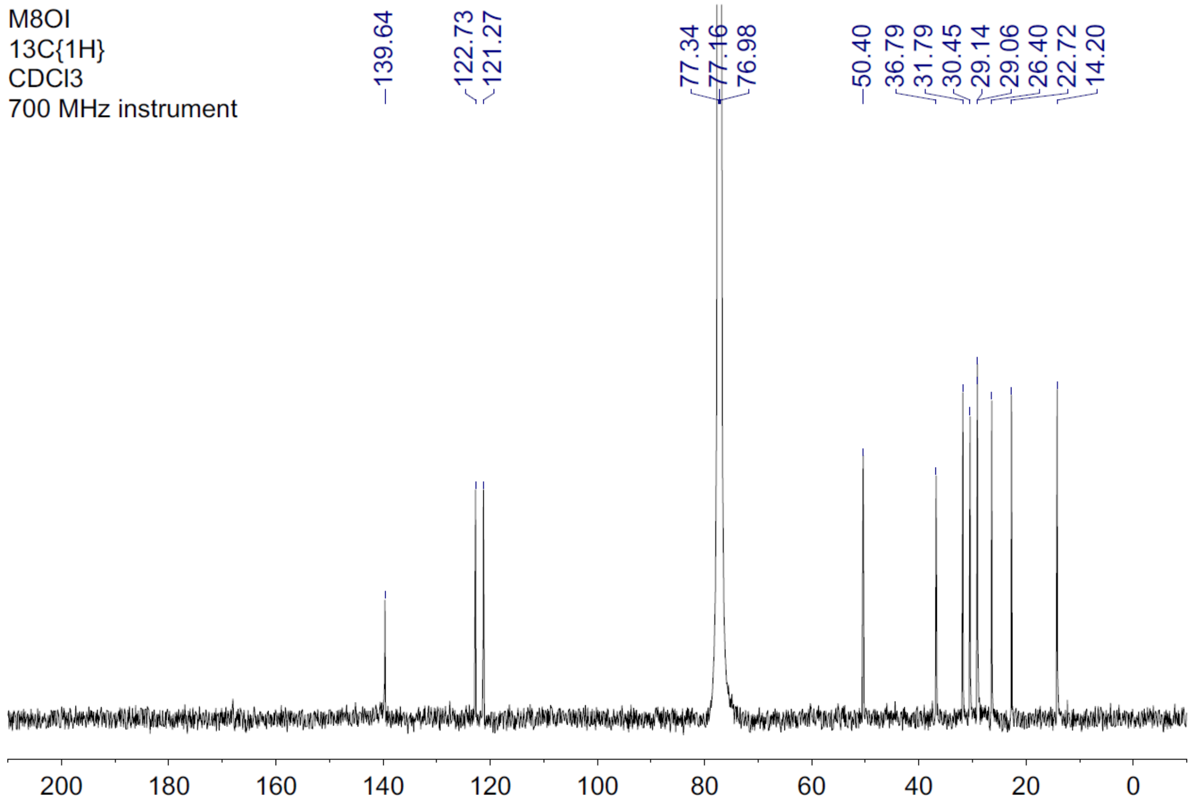
M8OI
13C{1H}
CDCl3
700 MHz instrument
139.64
122.73
121.27
77.34
77.16
76.98
50.40
36.79
31.79
30.45
29.14
29.06
26.40
22.72
14.20
200
180
160
140
120
100
80
60
40
20
0

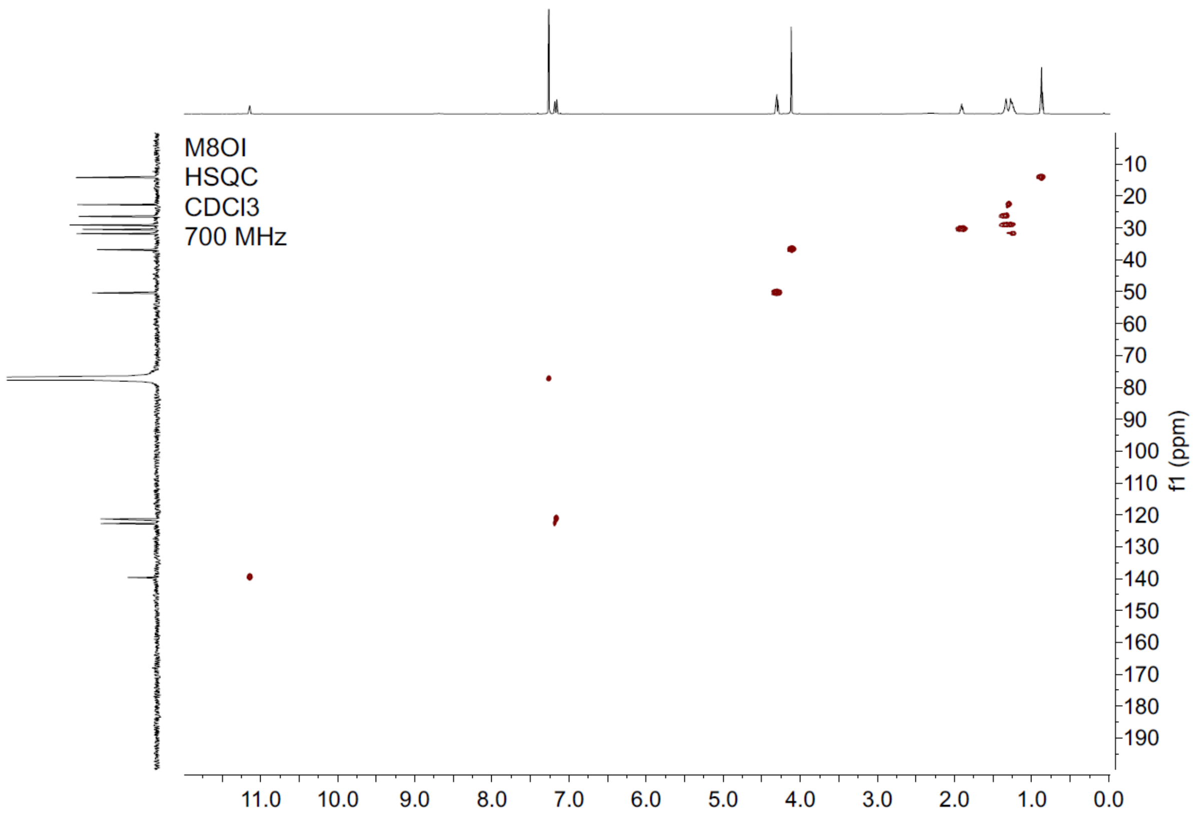
M8OI
HSQC
CDCl3
700 MHz
f1 (ppm)
11.0
10.0
9.0
8.0
7.0
6.0
5.0
4.0
3.0
2.0
1.0
0.0

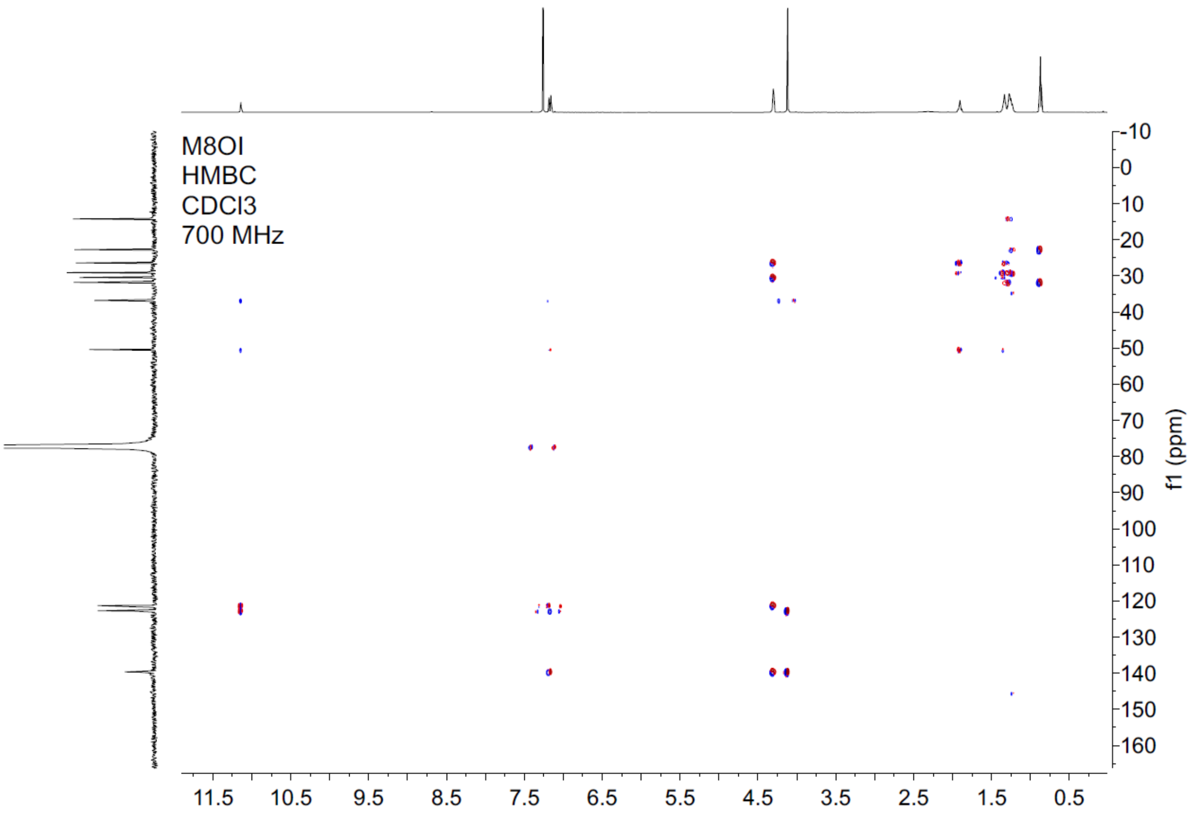
M8OI
HMBC
CDCl3
700 MHz
f1 (ppm)
-10
0
10
20
30
40
50
60
70
80
90
100
110
120
130
140
150
160
11.5
10.5
9.5
8.5
7.5
6.5
5.5
4.5
3.5
2.5
1.5
0.5

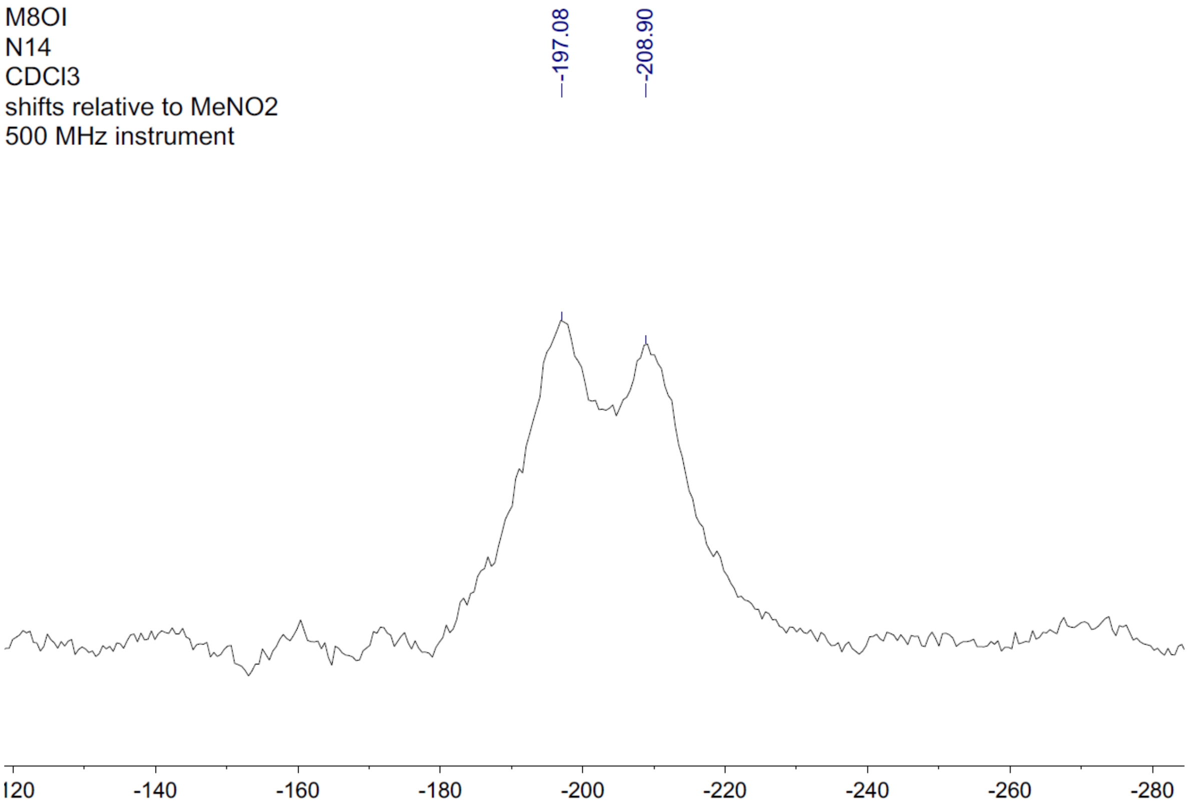
M8OI
N14
CDCl3
shifts relative to MeNO2
500 MHz instrument
-197.08
-208.90
-120
-140
-160
-180
-200
-220
-240
-260
-280

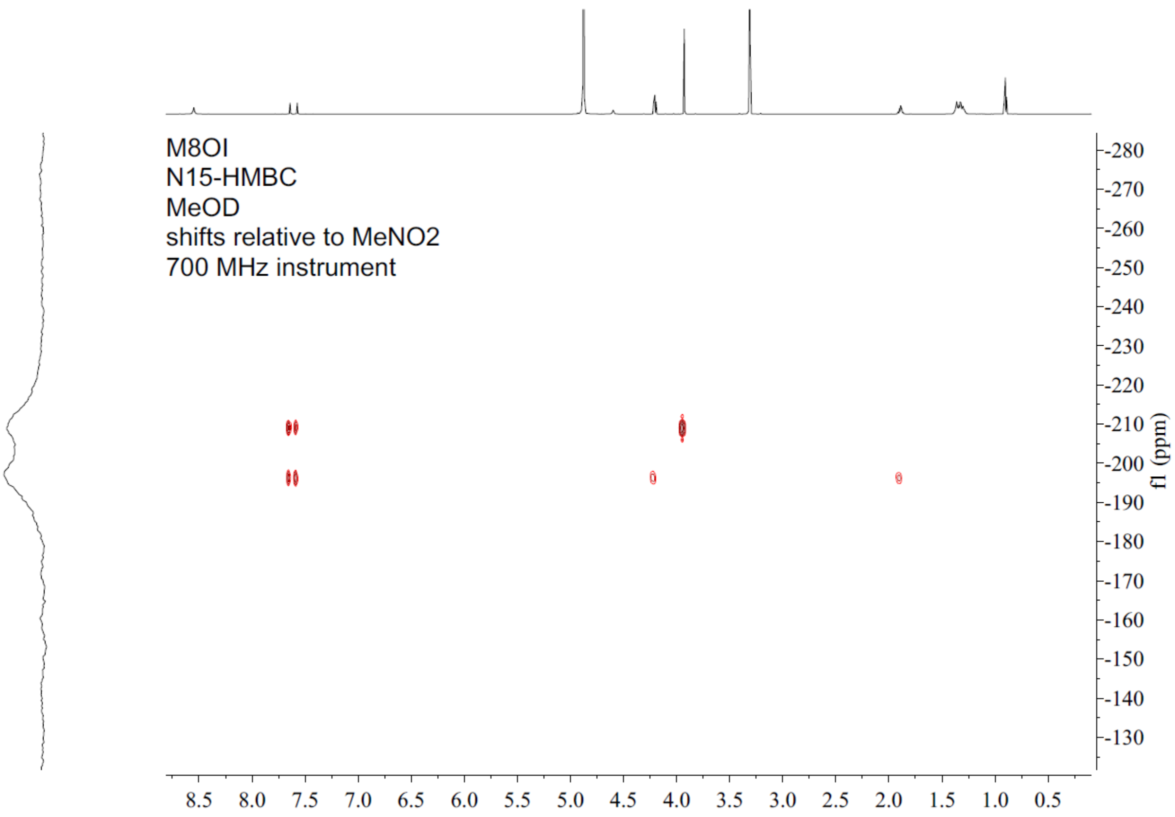
M8OI
N15-HMBC
MeOD
shifts relative to MeNO2
700 MHz instrument
f1 (ppm)

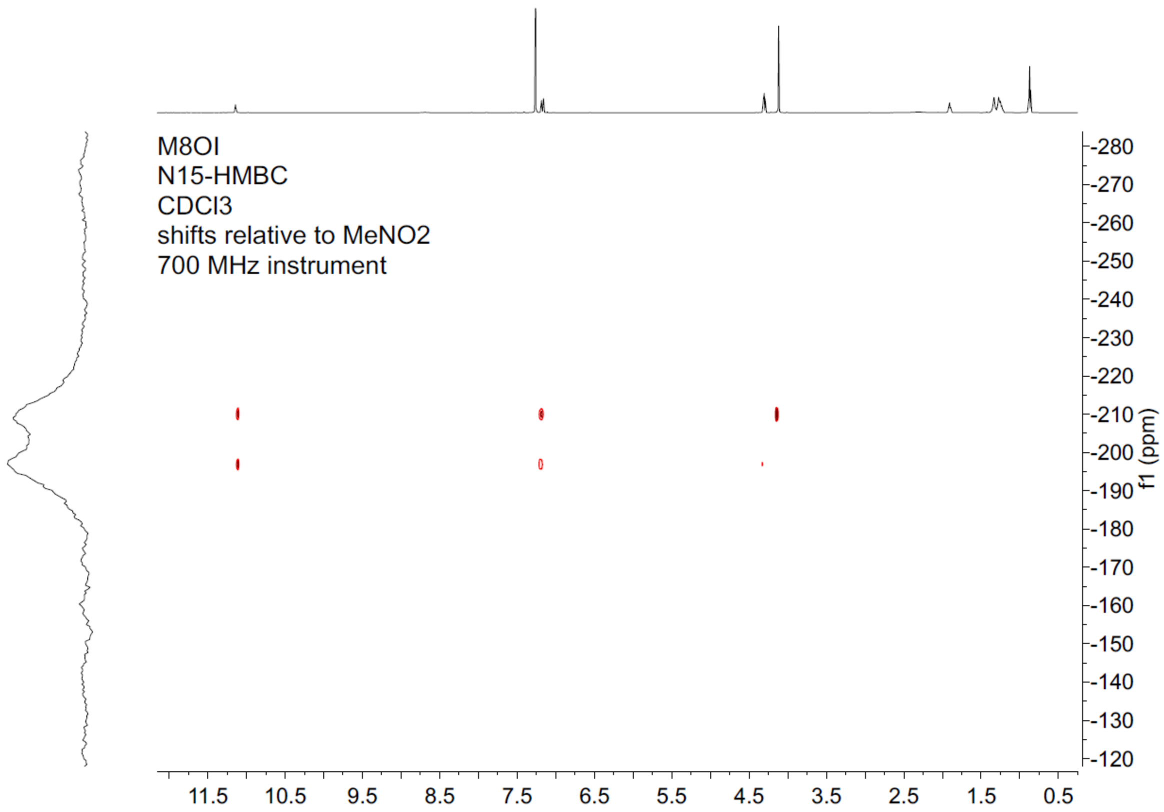
M8OI
N15-HMBC
CDCl3
shifts relative to MeNO2
700 MHz instrument
f1 (ppm)

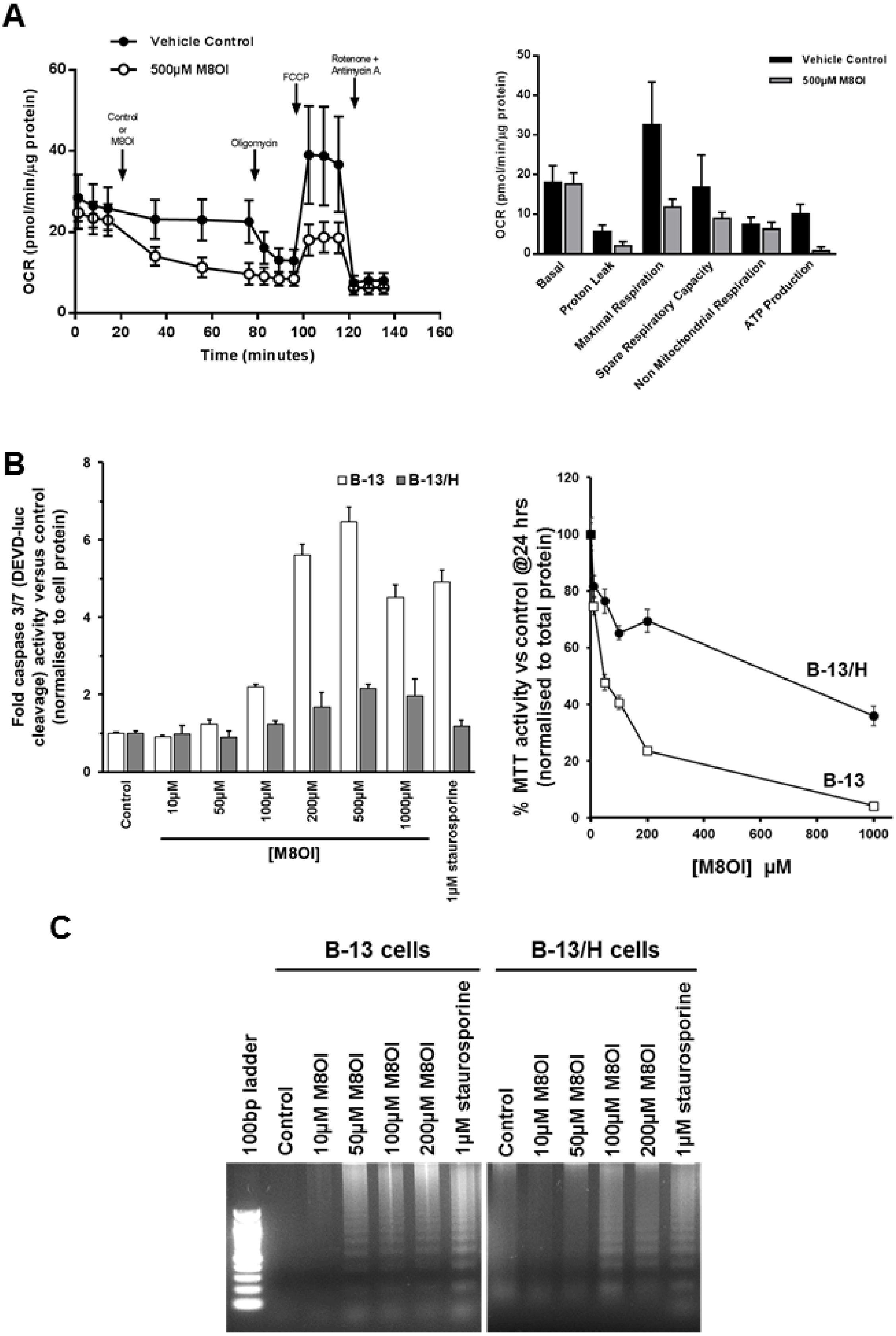
A
Vehicle Control
500µM M8OI
OCR (pmol/min/µg protein)
Time (minutes)
Control or M8OI
Oligomycin
FCCP
Rotenone + Antimycin A
Basal
Proton Leak
Maximal Respiration
Spare Respiratory Capacity
Non Mitochondrial Respiration
ATP Production
B
B-13
B-13/H
Fold caspase 3/7 (DEVD-luc cleavage) activity versus control (normalised to cell protein)
Control
10µM
50µM
100µM
200µM
500µM
1000µM
1µM staurosporine
[M8OI]
% MTT activity vs control @24 hrs (normalised to total protein)
[M8OI] µM
C
B-13 cells
B-13/H cells
100bp ladder
Control
10µM M8OI
50µM M8OI
100µM M8OI
200µM M8OI
1µM staurosporine

Fig. S8. B-13 cells are more sensitive to M8OI than the epthelial B-13/H cell. A, left panel, seahorse timecourse study with B-13/H cells; right panel, data are the mean and SD of 6 readings from the same experiment, typical of at least 5 separate experiments. **B**, left panel, caspase 3/7 activity in B-13 or B-13/H cells after 18 hours exposure, expressed relative to their respective control cells. Data are the mean and SD of 3 separate determinations from the same experiment, typical of at least 8 separate experiments; right panel, effect of M80I (chloride salt) on MTT activity in B-13 or B-13/H cells after 24 hours, expressed relative to their respective control treated cells. Data are the mean and SD of 3 separate treatments for the same experiment typical of at least 3 separate experiments. **C**, B-13 or B-13/H cells were treated with the indicated concentration of M8OI or staurosporine for 24 hours prior to genomic DNA isolation and analysis for nucleosomal ladder formation. Data are typical of at least 3 separate experiments.

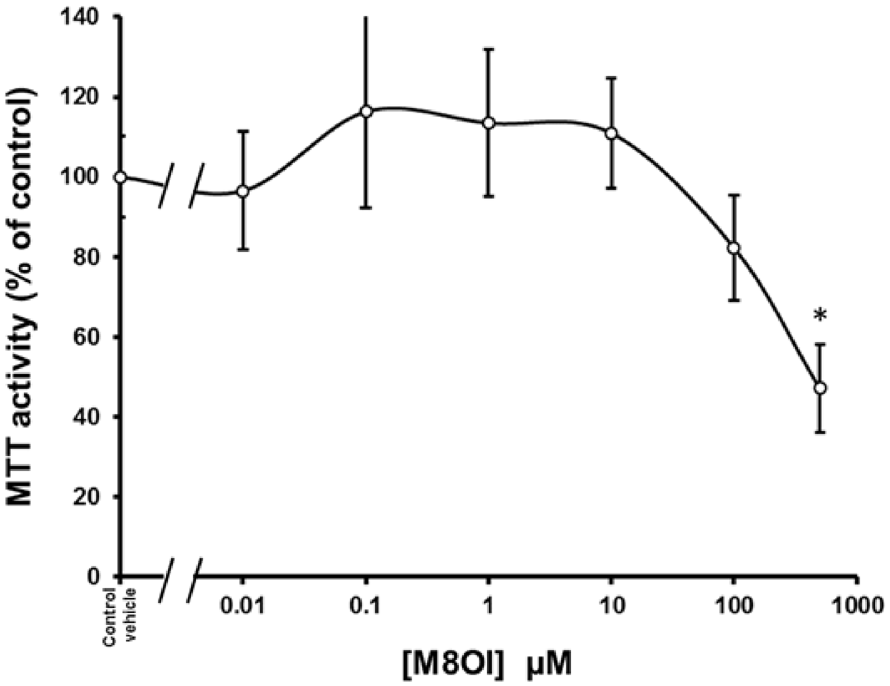

Fig. S9. M8OI is mildly toxic to human cholangiocyte-like H69 cells. MTT reduction following 24 hour exposure with the indicated concentration of M8OI. Data are the mean and SD of 6 separate determinations and are expressed as a percentage of 0.1% (v/v) solvent control vehicle. *Significantly different MTT activity from solvent control ($p < 0.05$) using ANOVA/Bonferroni-Holm comparison between groups.

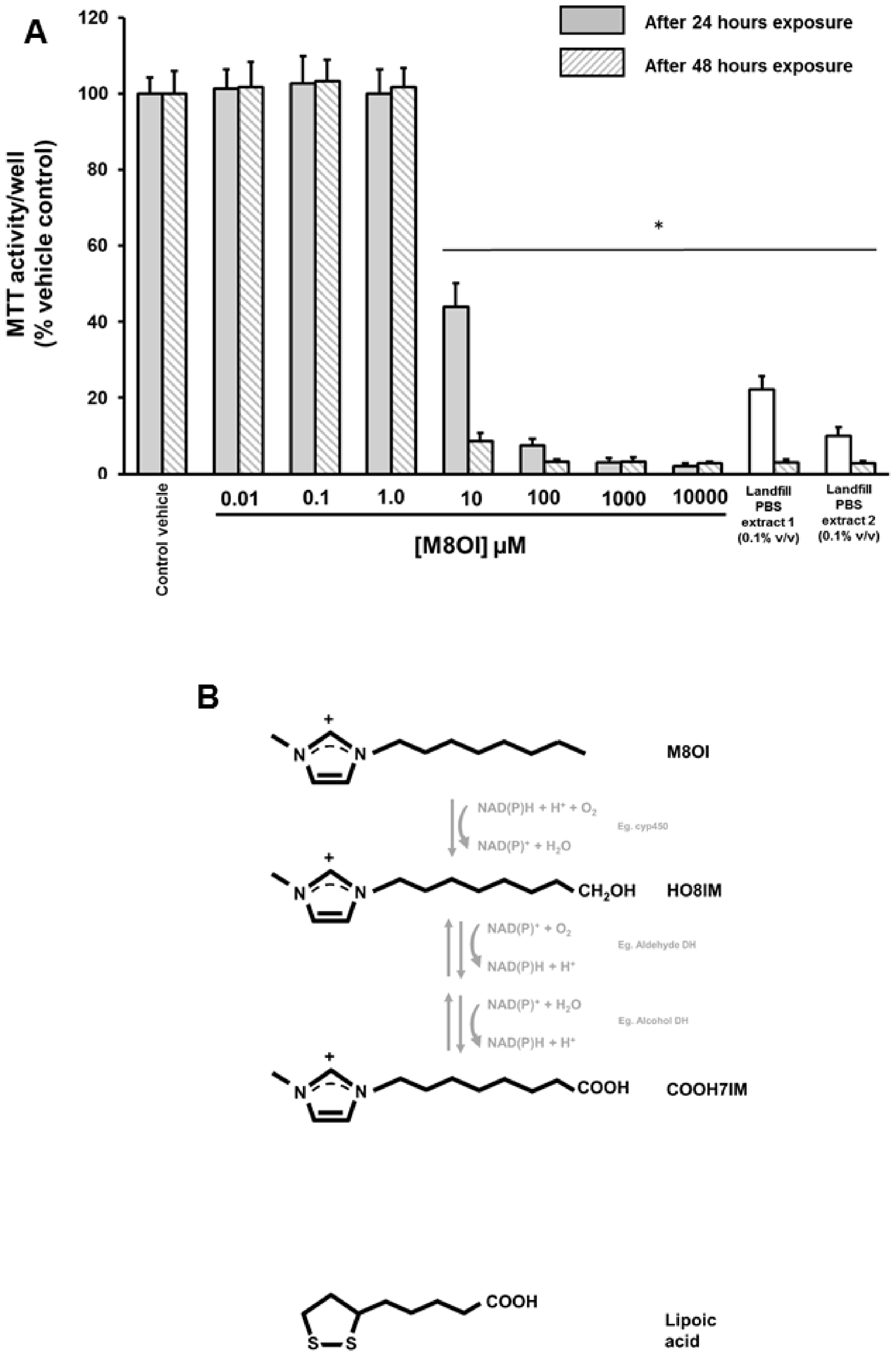

Fig. S10. Effect of M8OI on B-13 cells (MTT activity) and proposed metabolism of M8OI to a carboxylic acid and structural comparison to lipoic acid. **A**, MTT reduction in B-13 cells after exposure

to M8OI or the indicates PBS extracts for the indicated time. Data are the mean and SD of 3 separate determinations and are expressed as a percentage of 0.1% (v/v) solvent control vehicle. *Significantly different MTT activity from solvent control ($p < 0.05$) using ANOVA/Bonferroni-Holm comparison between groups. **B**, M8OI, 3-methyl-1-octyl-1H-imidazol-3-ium; HO8IM, 1-(8-hydroxyoctyl)-3-methyl-imidazolium; COOH7IM, 1-(7-carboxyheptyl)-3-methyl-1H-imidazol-3-ium.

A

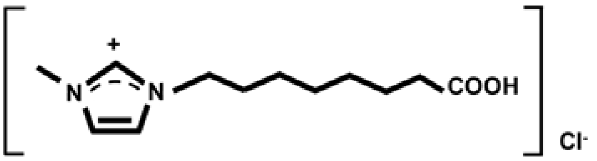

1-(7-carboxyheptyl)-3-methyl-1H-imidazol-3-ium chloride
(COOH7IM)

Formula / Mol. Wt.: C12H21ClN2O2 / 260.76 g mol[-1]

B HPLC – ELSD detection

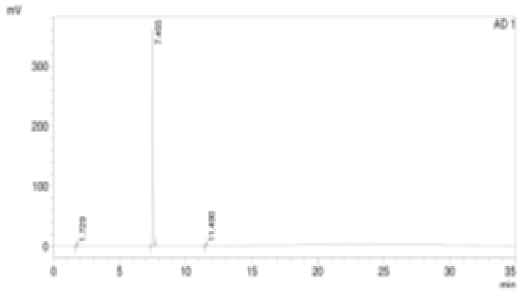

C MS- ESI+, predicted 226 Da fragment [C12H21N2O2 + H]$^+$

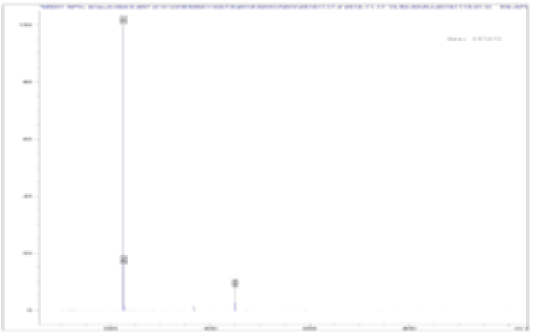

D ^{1}H NMR spectrum

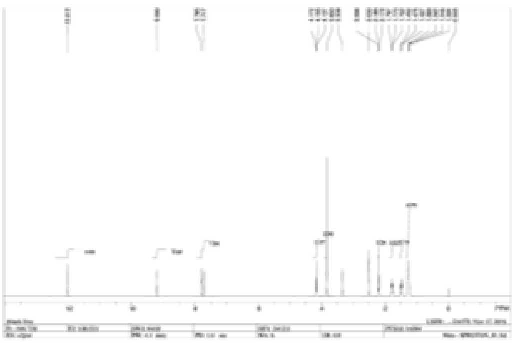

E ^{13}C NMR spectrum (D2O)

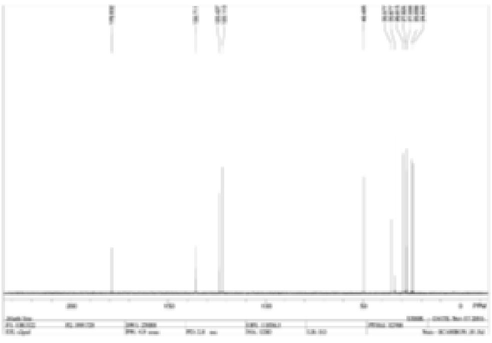

Fig. S11. A, Analytical data for the synthesised COOH7IM.

Table S1. Total metal analysis in soil samples.

	Sample ID	Metal (mg/kg oven dried soil) Sb	As	B	Ba	Cd	Co	Cr	Cu	Pb	Mn	Hg	Mo	Ni	Se	Zn
Samples in close proximity to waste tip A and B	1	12.4	12.1	10.0	578	16.7	15.6	28.9	201	409	636	0.92	2.50	44.4	0.88	506
	2	5.51	12.1	14.2	177	0.98	9.03	10.7	50.8	260	633	<0.3	3.21	30.1	<0.3	154
	3	6.83	5.34	2.95	228	0.32	13.0	17.9	29.7	80.6	1000	<0.3	1.08	26.4	<0.3	148
	4	4.17	9.34	8.05	375	1.34	44.9	21.4	45.2	64.5	12890	<0.3	2.12	82.9	6.04	221
	5	13.3	5.61	19.1	299	0.78	9.84	28.0	128	237	510	<0.3	2.88	24.2	<0.3	284
	6	4.34	3.79	3.03	242	0.72	8.00	12.2	22.0	77.3	492	<0.3	0.96	16.8	<0.3	94.4
	7	3.31	2.95	4.69	197	0.34	6.89	13.2	27.0	57.2	379	<0.3	1.21	14.9	<0.3	100
	8	1.68	3.24	11.6	161	0.34	6.02	18.5	28.7	39.5	483	<0.3	1.19	12.0	<0.3	123
	9	4.38	10.1	5.35	260	0.55	11.7	16.6	45.6	124	739	<0.3	1.34	22.1	<0.3	160
	10	8.91	7.29	6.71	228	0.63	11.0	22.8	63.8	172	544	<0.3	1.77	26.1	<0.3	224
	11	8.32	4.06	11.9	264	0.64	7.33	20.8	62.4	172	416	<0.3	2.54	19.5	<0.3	222
	12	3.35	6.09	15.2	279	0.56	44.1	18.3	147	114	740	<0.3	1.69	22.8	<0.3	492
	13	2.36	3.80	7.99	263	0.39	9.03	13.5	30.2	71.2	505	<0.3	1.08	16.2	<0.3	125
**Upper 95% confidence limit**		**8.182**	**8.488**	**12.12**	**334.3**	**4.425**	**22.73**	**21.93**	**99.64**	**204.9**	**3491**		**2.250**	**38.19**	**1.912**	**297.3**
	Mean ± SD	6 ± 4	7 ± 3	9 ± 5	273 ± 107	2 ± 4	15 ± 13	19 ± 6	68 ± 56	144 ± 105	1536 ± 3416	0 ± 0	2 ± 1	28 ± 19	3 ± 4	219 ± 136
Control samples	1	104	12.9	4.14	208	0.78	10.1	25.6	56.3	157	410	<0.3	2.78	22.4	<0.3	177
	2	41.1	6.63	4.48	70.1	<0.30	10.3	35.1	21.0	42.9	570	<0.3	1.54	16.3	<0.3	78.3
	3	6.85	14.3	5.01	382	0.95	18.4	26.1	85.9	779	2388	1.55	2.34	41.5	<0.3	223
	Mean ± SD	51 ± 49	11 ± 4	5 ± 0	220 ± 156	1 ± 1	13 ± 5	29 ± 5	54 ± 32	326 ± 396	1122 ± 1098	1 ± 1	2 ± 1	27 ± 13	0 ± 0	160 ± 74
SGV$	Residential<br>allotment<br>commercial	-<br>-<br>-	32<br>43<br>640	-<br>-<br>-	-<br>-<br>-	10<br>1.8<br>230	-<br>-<br>-	45<br>45<br>-	-<br>-<br>-	290<br>250<br>-	-<br>-<br>-	1*<br>26*<br>26*	-<br>-<br>-	130<br>230<br>1800	350<br>120<br>13000	-<br>-<br>-

$SGV, Soil Guideline Values. https://www.gov.uk/government/publications/land-contamination-soil-guideline-values-sgvs. SGVs and the framework documents provide scientifically based information on the assessment of risks to human health from land contamination. They provide non-statutory technical guidance to regulators and their advisors in support of the statutory regimes addressing land contamination, particularly Part 2A of the Environmental Protection Act 1990 and the consideration of land affected by contamination under the Town and Country Planning Acts.

*elemental values – inorganic Hg2+ levels 170, 80 and 3600 mg/kg; methyl Hg^{4+} levels 11, 8 and 410 mg/kg respectively for residential, allotment and commercial land soils.

Values highlighted in red > 95% limit high range for all tip samples.

Table S2. PAH analysis in soil samples.

		PAH (mg/kg soil)									
	Sample ID	Anthraquinone	Anthracene	Phenanthrene	Naphthalenes	Chrysene + benzanthracene	Idenopyrene + dibenzoanthracene + perylene	Bifluoranthene + benzoperylene + Perlene	Fluorenes	Pyrene + fluoranthene	Biphenyls
Samples in close proximity to landfill	1	n/d	0.026	0.103	0.077	0.269	0.125	0.374	0.054	0.516	0.103
	2	0.008	0.105	0.183	0.236	0.949	0.436	1.433	0.092	1.888	0.183
	3	n/d	1.181	1.701	0.437	3.843	2.230	6.220	0.570	11.473	1.701
	4	n/d	0.047	0.061	0.143	0.284	0.196	0.489	0.028	0.628	0.061
	5	n/d	0.132	0.088	0.107	0.677	0.526	1.104	0.044	1.366	0.088
	6	n/d	0.013	0.020	0.029	0.176	0.123	0.305	0.011	0.325	0.020
	7	0.008	0.164	0.193	0.187	0.958	0.697	1.812	0.085	2.187	0.193
	8	n/d	0.089	0.096	0.099	0.875	0.422	1.799	0.051	1.649	0.096
	9	0.019	2.174	2.187	0.365	11.854	6.013	17.845	0.940	27.579	2.187
	10	n/d	0.578	0.285	0.215	1.658	1.266	3.242	0.134	3.537	0.285
	11	n/d	0.109	0.117	0.470	0.905	0.635	1.626	0.054	1.948	0.117
	12	0.014	0.654	0.666	0.196	1.806	1.063	2.859	0.266	4.519	0.666
	13	n/d	0.047	0.276	0.062	1.968	0.990	2.781	0.127	4.277	0.276
	Mean ± SD	0.0038 ± 0.0065	0.409 ± 0.632	0.460 ± 0.686	0.202 ± 0.142	2.02 ± 3.11	1.13 ± 1.58	3.22 ± 4.67	0.189 ± 0.270	4.76 ± 7.45	0.460 ± 0.686
Control samples	1 (PL)	0.110	0.037	0.624	0.054	0.363	0.003	0.604	0.038	0.760	0.033
	2 (Ben)	nd	1.82	21.02	0.205	12.547	9.494	25.17	0.516	21.02	0.407
	3 (Mor)	nd	1.68	27.84	0.763	11.425	7.965	20.794	1.168	27.84	0.964
	Mean ± SD	n/a	1.179 ± 0.991	16.495 ± 14.161	0.341 ± 0.373	8.112 ± 6.734	5.821 ± 5.096	15.523 ± 13.014	0.574 ± 0.567	16.540 ± 14.085	0.468 ± 0.468

n/d, not detected; n/a, not applicable. Total mean PAHs in samples in close proximity to landfill site, 13 ± 19 mg/kg soil; total mean PAHs in control soil samples, 65 ± 54 mg/kg soil.

Table S3. Analysis for 22 pesticides in the soil samples.

		Pesticide (ppm of extract, equivalent to 60g soil)										
	Sample ID	Ametryn	Terbutryn	Cyanazine	Atrazine	Simetryn	Spiroxamine	Kresoxim methyl	Azoxystrobin	Dimethomorph	Pyraclostrobin	Chlortoluron
Soil samples in close proximity to landfill site	1	0.00	0.02	0.01	0.19	0.00	0.25	0.00	0.03	0.01	0.02	0.04
	2	0.01	0.00	0.00	0.02	0.00	0.01	0.00	0.00	0.00	0.00	0.00
	3	0.08	0.03	0.01	0.28	0.01	0.23	0.44	0.04	0.00	0.03	0.04
	4	0.04	0.01	0.00	0.02	0.00	0.06	0.10	0.01	0.00	0.01	0.04
	5	0.01	0.00	0.00	0.00	0.00	0.03	0.00	0.00	0.00	0.00	0.01
	6	0.02	0.00	0.00	0.02	0.00	0.06	0.00	0.02	0.00	0.01	0.01
	7	0.00	0.00	0.00	0.03	0.00	0.02	0.00	0.00	0.00	0.00	0.01
	8	0.00	0.00	0.02	0.08	0.00	0.19	0.00	0.13	0.01	0.03	0.05
	9	0.00	0.01	0.01	0.27	0.00	0.18	0.00	0.12	0.00	0.04	0.04
	10	0.01	0.02	0.01	0.01	0.00	0.18	0.09	0.00	0.00	0.05	0.01
	11	0.03	0.03	0.00	0.13	0.00	0.03	0.01	0.04	0.00	0.01	0.01
	12	0.00	0.02	0.01	0.09	0.00	0.18	0.75	0.03	0.01	0.05	0.03
	13	0.00	0.00	0.00	0.01	0.00	0.02	0.06	0.01	0.00	0.01	0.00
	Mean ± SD	0.02 ± 0.023	0.01 ± 0.011	0.01 ± 0.006	0.09 ± 0.099	0.00 ± 0.003	0.11 ± 0.091	0.11 ± 0.226	0.03 ± 0.043	0.00 ± 0.004	0.02 ± 0.018	0.02 ± 0.017
Control soil samples	1	0.00	0.02	0.01	0.26	0.00	0.15	0.35	0.06	0.01	0.05	0.04
	2	0.00	0.02	0.00	0.00	0.00	0.15	0.00	0.01	0.00	0.05	0.04
	3	0.00	0.02	0.00	0.18	0.00	0.16	0.78	0.02	0.00	0.06	0.52
	Mean ± SD	0.00 ± 0.002	0.02 ± 0.002	0.00 ± 0.002	0.15 ± 0.135	0.00 ± 0.001	0.16 ± 0.005	0.38 ± 0.390	0.03 ± 0.028	0.00 ± 0.002	0.05 ± 0.005	0.20 ± 0.277
												$p < 0.02$

		Pesticide (ppm)										
	Sample ID	Siduron	Monuron	Monolinuron	Diuron	Dicrotophos	Chlorfenvinphos	Tetrachlorvinphos	Omethoate	Ethoprophos	Dimethoate	Azinphos methyl
Soil samples in close proximity to landfill site	1	0.06	0.06	0.00	1.95	0.00	0.00	0.02	0.91	0.28	0.02	0.05
	2	0.00	0.00	0.00	0.08	0.00	0.00	0.00	0.09	0.00	0.00	0.00
	3	0.00	0.18	0.00	1.99	0.00	0.00	0.00	1.12	0.02	0.00	0.00
	4	0.00	0.03	0.00	0.46	0.00	0.01	0.00	0.16	0.01	0.03	0.02
	5	0.00	0.01	0.00	0.20	0.00	0.01	0.00	0.03	0.00	0.00	0.00
	6	0.00	0.01	0.01	0.38	0.00	0.00	0.00	0.22	0.00	0.01	0.00
	7	0.00	0.02	0.00	0.18	0.00	0.01	0.00	0.05	0.01	0.00	0.00
	8	0.09	0.38	0.00	1.73	0.01	0.03	0.01	0.94	0.08	0.00	0.00
	9	0.00	0.06	0.00	1.19	0.00	0.08	0.02	0.68	0.02	0.00	0.03
	10	0.01	0.05	0.00	1.60	0.00	0.09	0.00	0.89	0.15	0.00	0.01
	11	0.00	0.03	0.00	0.39	0.00	0.00	0.00	0.05	0.01	0.01	0.00
	12	0.02	0.18	0.00	1.43	0.00	0.11	0.00	0.66	0.22	0.00	0.12
	13	0.00	0.01	0.00	0.18	0.00	0.02	0.00	0.06	0.01	0.00	0.00
	Mean ± SD	0.02 ± 0.029	0.08 ± 0.109	0.00 ± 0.003	0.90 ± 0.751	0.00 ± 0.002	0.03 ± 0.039	0.01 ± 0.008	0.45 ± 0.418	0.06 ± 0.093	0.01 ± 0.010	0.02 ± 0.034
Control soil samples	1	0.00	0.03	0.00	4.08	0.00	0.07	0.01	0.40	0.14	0.07	0.00
	2	0.00	0.02	0.00	1.57	0.00	0.03	0.00	0.20	0.03	0.00	0.01
	3	0.07	0.07	0.00	1.53	0.00	0.09	0.01	0.12	0.04	0.00	0.00
	Mean ± SD	0.02 ± 0.043	0.04 ± 0.024	0.00 ± 0.00	2.39 ± 1.458	0.00 ± 0.001	0.06 ± 0.031	0.01 ± 0.004	0.24 ± 0.145	0.07 ± 0.061	0.02 ± 0.043	0.00 ± 0.004
					$p < 0.02$							

REFERENCES

52. Wallace K, Marek CJ, Hoppler S, Wright MC. Glucocorticoid-dependent transdifferentiation of pancreatic progenitor cells into hepatocytes is dependent on transient suppression of WNT signalling. J Cell Sci. 2010; 123: 2103-10.

53. Probert PM, Chung GW, Cockell SJ, Agius L, Mosesso P, White SA, Oakley F, Brown CD, Wright MC. Utility of B-13 progenitor-derived hepatocytes inhepatotoxicity and genotoxicity studies. Toxicol Sci. 2014: 137: 350-70 (2014).

54. Fairhall EA, Charles MA, Wallace K, Schwab CJ, Harrison CJ, Richter M, Hoffmann SA, Charlton KA, Zeilinger K, Wright MC. The B-13 hepatocyte progenitor cell resists pluripotency induction and differentiation to non-hepatocyte cells. Toxicology Research 2013; 2: 308–320 (2013).

55. Grubman SA, Perrone RD, Lee DW, Murray SL, Rogers LC, Wolkoff LI, Mulberg AE, Cherington V, Jefferson DM. Regulation of intracellular pH by immortalized human intrahepatic biliary epithelial cell lines. Am J Physiol. 1994; 266: G1060-70.

56. Wright MC, Issa R, Smart DE, Trim N, Murray GI, Primrose JN, Arthur MJ, Iredale JP, Mann DA. Gliotoxin stimulates the apoptosis of human and rat hepatic stellate cells and enhances the resolution of liver fibrosis in rats. Gastroenterology 2001; 121: 685-698.

57. Meyer SK, Probert PM, Lakey AK, Blake LI, Leitch A, Jowsey PA, Cooke MP, Blain PG, Wright MC. Environmental xenoestrogens super-activate a variant murine ER beta in cholangiocytes. Toxicol Sci. 2017; 156: 54-71.

58. Probert PM, Palmer JM, Alhusainy W, Amer AO, Rietjens IM, White SA, Jones DE, Wright MC. Progenitor-derived hepatocyte-like (B-13/H) cells metabolise 1:-hydroxyestragole to a genotoxic species via a SULT2B1-dependent mechanism. Toxicol Lett 2016; 243: 98-110.

59. Harvey JL, Paine AJ, Maurel P, Wright MC. Effect of the adrenal 11-beta-hydroxylase inhibitor metyrapone on human hepatic cytochrome P-450 expression: induction of cytochrome P-450 3A4. Drug Metab Dispos 2000; 28: 96-101.

60. Kendrick SF, O:Boyle G, Mann J, Zeybel M, Palmer J, Jones DE, Day CP. Acetate, the key modulator of inflammatory responses in acute alcoholic hepatitis. Hepatology 2010; 51: 1988-97.

61. Axon A, May FE, Gaughan LE, Williams FM, Blain PG, Wright MC. Tartrazine and sunset yellow are xenoestrogens in a new screening assay to identify modulators of human oestrogen receptor transcriptional activity. Toxicology 2012; 298: 40-51.

62. Wallace K, Flecknell PA, Burt AD, Wright MC. Disrupted pancreatic exocrine differentiation and malabsorption in response to chronic elevated systemic glucocorticoid. Am J Pathol. 2010; 177: 1225-32.

63. Eden PA, Schmidt TM, Blakemore RP, Pace NR. Phylogenetic analysis of Aquaspirillum magnetotacticum using polymerase chain reaction-amplified 16S rRNA-specific DNA. Int J Syst Bacteriol. 1991; 41: 324-5.

64. Wallace K, Cowie DE, Konstantinou DK, Hill SJ, Tjelle TE, Axon A, Koruth M, White SA, Carlsen H, Mann DA, Wright MC. The PXR is a drug target for chronic inflammatory liver disease. J Steroid Biochem Mol Biol. 2010; 120: 137-48.

65. EFSA Scientific Committee; Guidance on selected default values to be used by the EFSA Scientific Committee, Scientific Panels and Units in the absence of actual measured data. EFSA Journal 2012; 10: 2579.

66. Marek CJ, Tucker SJ, Konstantinou DK, Elrick LJ, Haefner D, Sigalas C, Murray GI, Goodwin B, Wright MC. Pregnenolone-16alpha-carbonitrile inhibits rodent liver fibrogenesis via PXR (pregnane X receptor)-dependent and PXR-independent mechanisms. Biochem J. 2005; 387: 601-8.

67. Green S. PPAR: a mediator of peroxisome proliferator action. Mutat Res. 1995;333:101-9.
